# Supplementary material for: Gene Protein Sequence Evolution Can Predict the Rapid Divergence of Ovariole Numbers in the Drosophila melanogaster Subgroup
Source: Genome Biol Evol. 2024 Jun 7;16(7):evae118. doi: 10.1093/gbe/evae118 (PMC11272079; doi:10.1093/gbe/evae118)
Supplement: evae118_Supplementary_Data [file evae118_supplementary_data.pdf]

Supplementary Material

Gene protein sequence evolution can predict the rapid divergence of ovariole numbers in the *Drosophila melanogaster* subgroup

Carrie A. Whittle, Cassandra G. Extavour

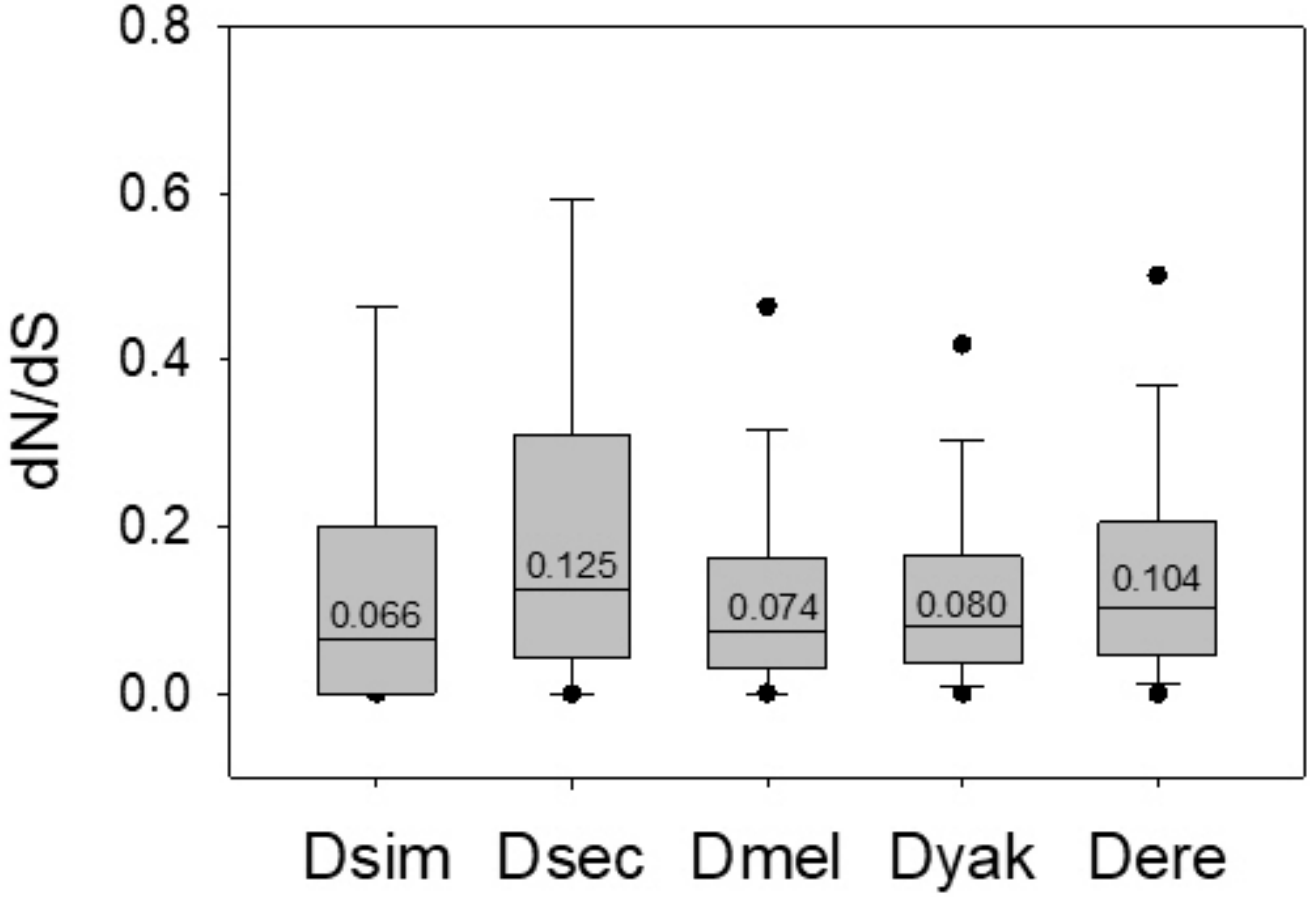

Figure S1. Box plots of the distribution of the free-ratio dN/dS values in each species terminal branch for all genes per genome with five-species orthologs in the *melanogaster* subgroup (Dsim = *Drosophila simulans*; Dsec = *D. sechellia*, Dmel = *D. melanogaster*, Dyak = *D. yakuba*, Dere = *D. erecta*). The median dN/dS value is shown within each box. Outliers are excluded for Dsim and Dsec as they were outside the range of the Y axis.

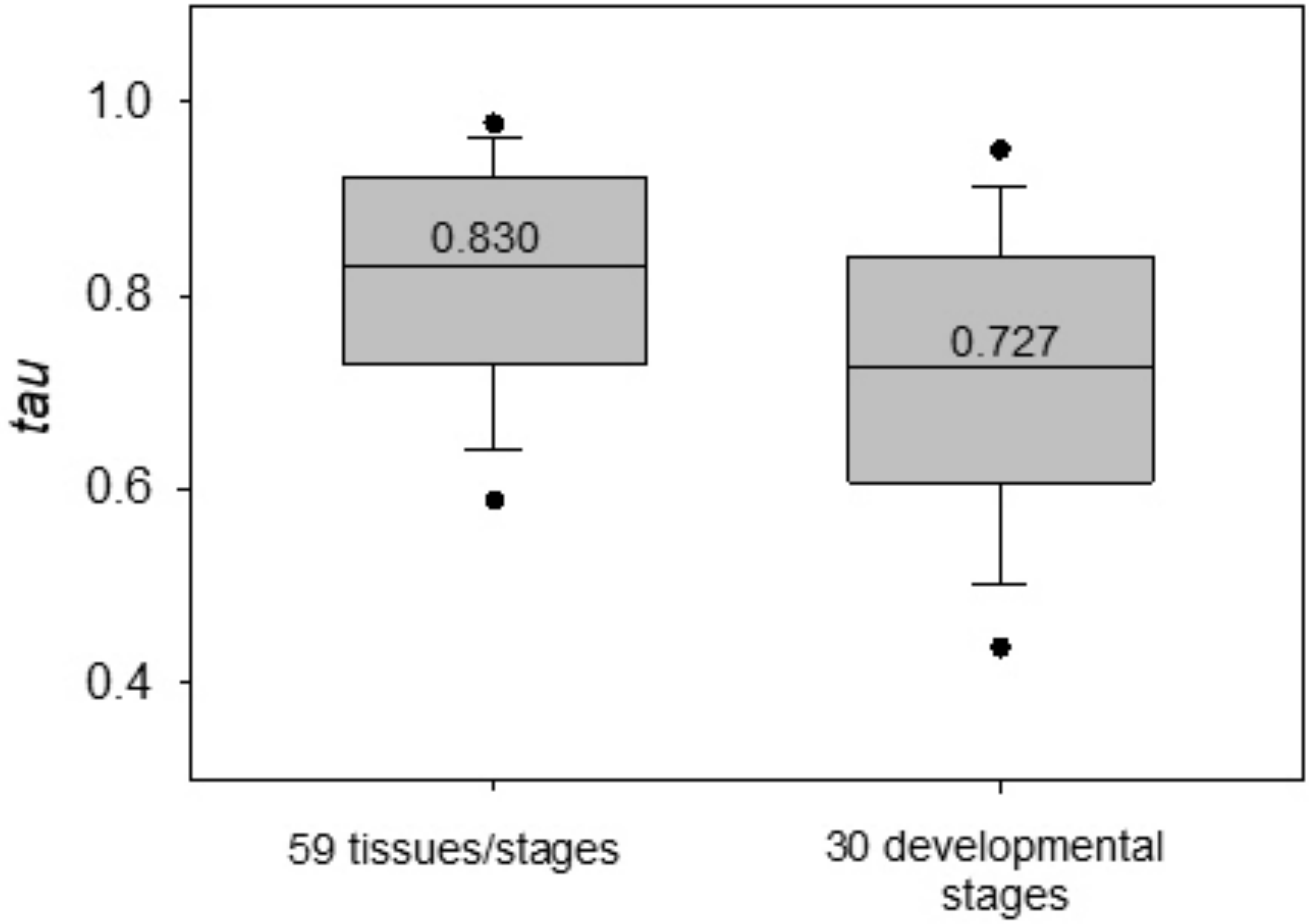

Figure S2. Box plots of the *tau* values across all studied genes in *D. melanogaster* using expression data across 59 tissues and developmental stages (combined) and solely using the data from 30 developmental stages. Values are for all genes with orthologs among the five studied species in the *melanogaster* subgroup. Median values are shown within bars. Tissues and stages are described in table S1.

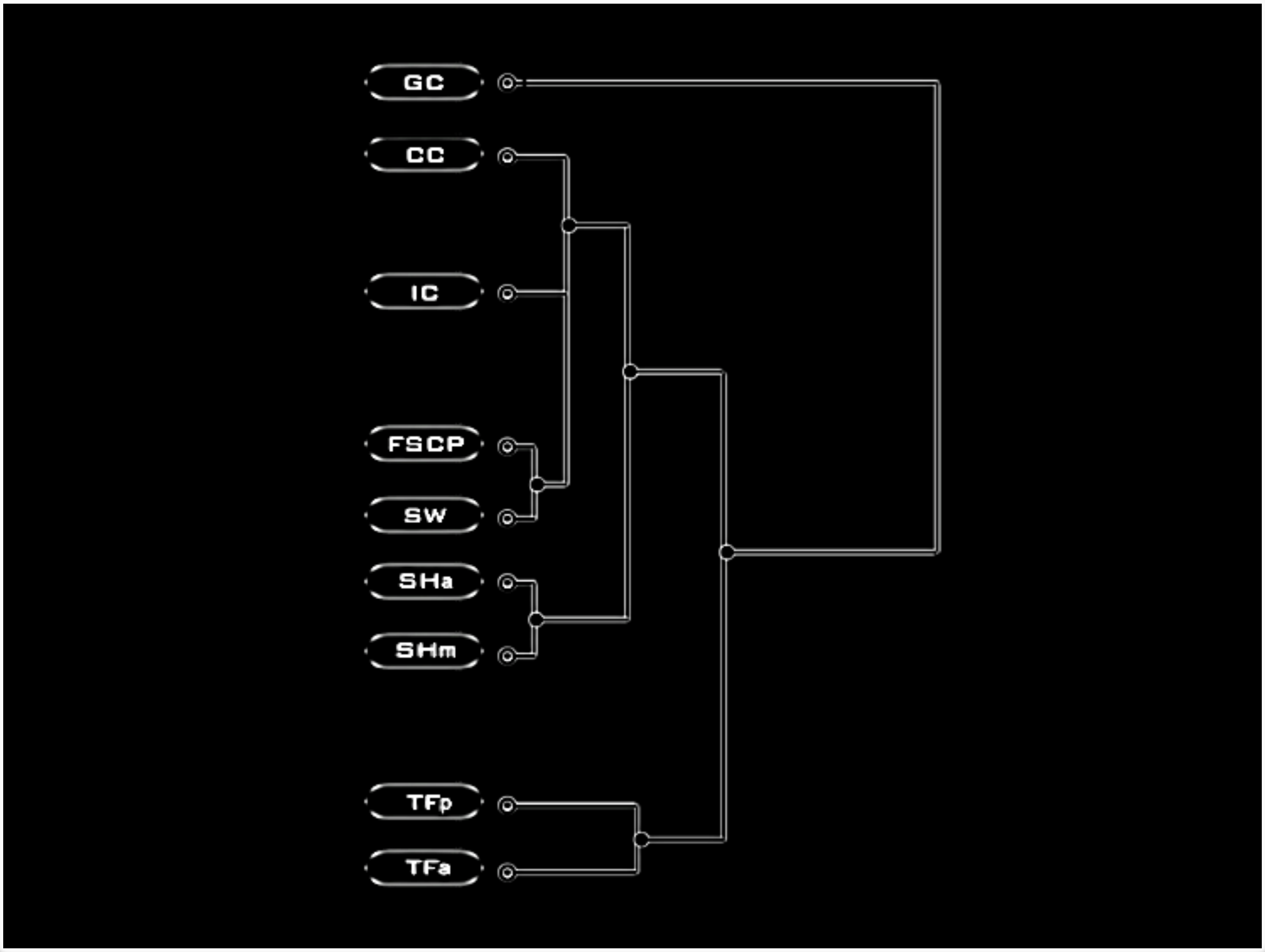

Figure S3. Clustering of the average standardized expression of all genes in the *D. melanogaster* genome (with non-zero expression) in the various cell types obtained from the study of LL3 larval ovaries in Slaidina, et al. (2020). The expression was clustered using hierarchical clustering and average linkage in the Morpheus program (see Materials and Methods). CC=cap cells, FSCP=follicle stem cell precursors, GC=germ cells, IC=intermingled cells, SHa= anterior sheath cells, SHm= migrating sheath cells, SW=swarm cells, TFa= anterior terminal filament cells, TFp= posterior terminal filament cells.

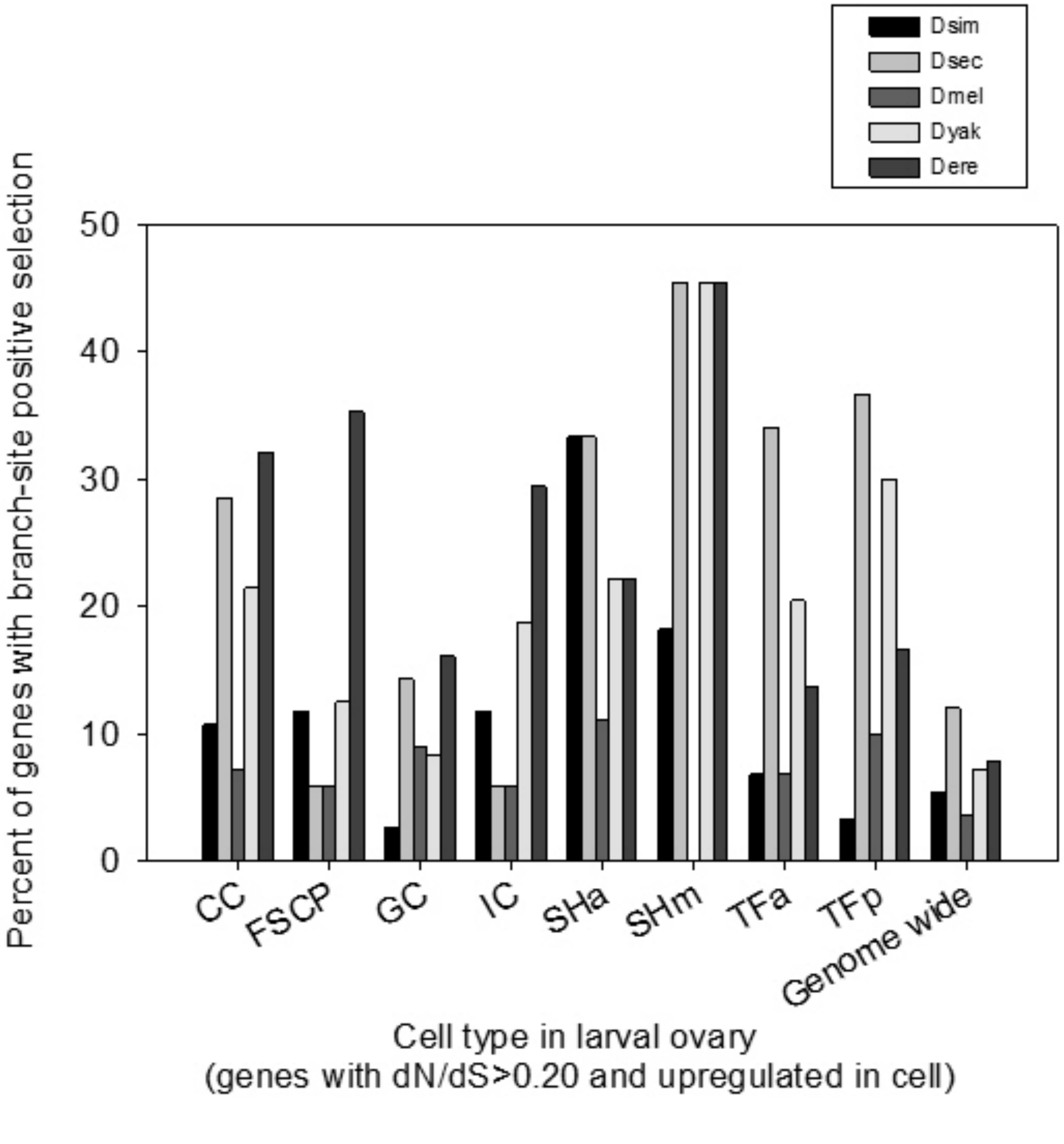

Figure S4. The percentage of the genes upregulated in a particular cell type using *D. melanogaster* sc-RNA seq(Slaidina, et al. 2020) and rapidly evolving in the *melanogaster* subgroup (M0 dN/dS>0.20) that exhibited branch-site positive selection in the *D. simulans* (Dsim), *D. sechellia (*Dsec), *D. melanogaster* (Dmel), *D. yakuba* (Dyak) or *D. erecta* (Dere) branches. The number of genes per category were as follows: CC (28), FSCP (17), GC (112), IC (17), SHa (9), SHm (11), SW (4), TFa (44), TFp (30). SW cells were excluded as too few genes were rapidly evolving for study. Note that a gene could be upregulated in more than one cell type. The genome-wide values are for all genes with orthologs in the *melanogaster* subgroup.

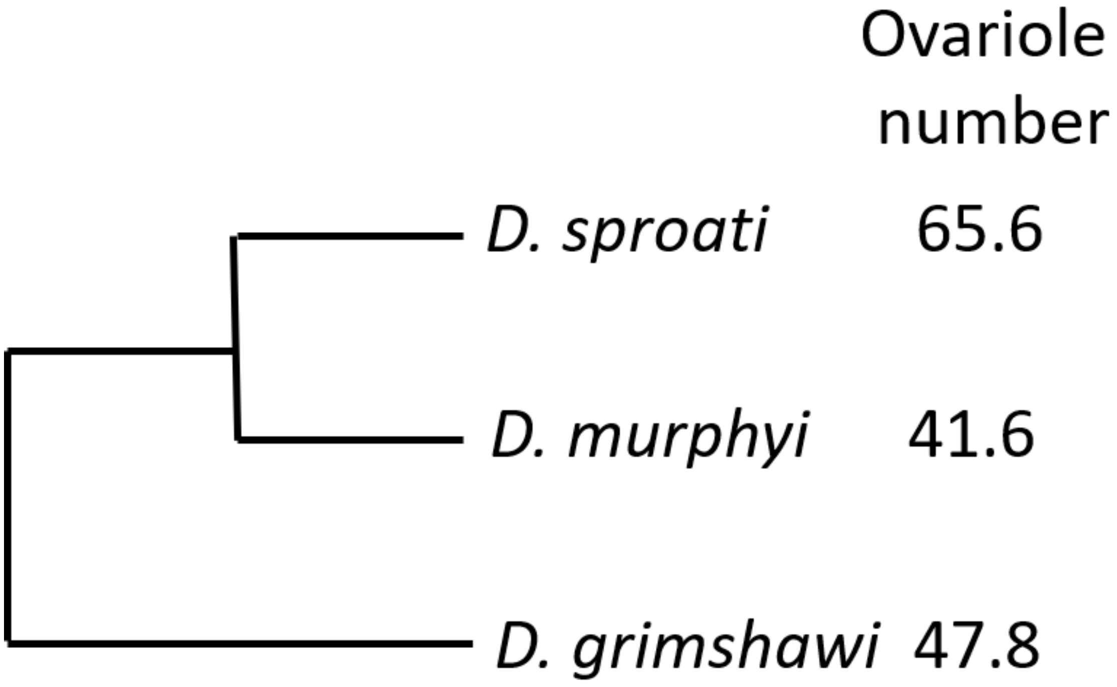

Figure S5. The phylogenetic relationship of the three Hawaiian *Drosophila* species under study and their mean ovariole numbers per female (phylogeny from (Kim, et al. 2021; Suvorov, et al. 2022), ovariole numbers from (Starmer, et al. 2003; Sarikaya, et al. 2019).

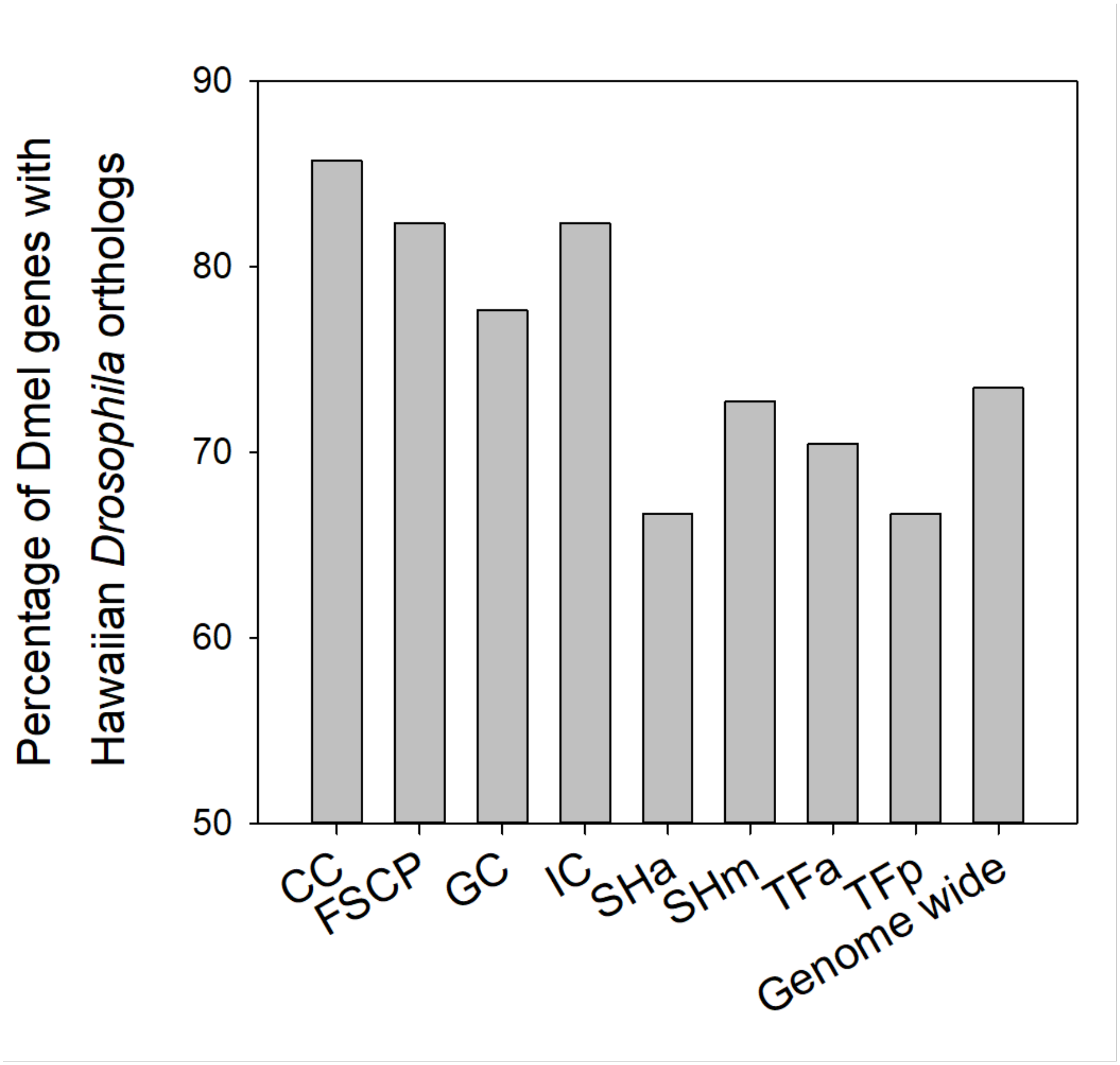

Figure S6. The percent of studied *D. melanogaster* sc-RNA seq genes upregulated and rapidly evolving in *D. melanogaster* that had three-species orthologs in Hawaiian *Drosophila*. N values in *D. melanogaster* are in Fig. 4. To be defined as having an orthologous gene set, the *D. melanogaster* gene had to have a match to the annotated genome *D. grimshawi*, and the three Hawaiian species had to all have high confidence orthologs to each other.

Table S1. The 30 developmental stages and 29 tissue types used for expression breadth analysis, or *tau*. The RPKM data is from ModEncode (Li, et al. 2014) and is available at FlyBase (Gramates, et al. 2022). Downloaded in April 2022.

**Developmental stages**	**Tissue types**
em0-2hr	A_MateF_1d_head
em2-4hr	A_MateF_4d_ovary
em4-6hr	A_MateM_1d_head
em6-8hr	A_VirF_1d_head
em8-10hr	A_VirF_4d_head
em10-12hr	A_MateF_20d_head
em12-14hr	A_MateF_4d_head
em14-16hr	A_MateM_20d_head
em16-18hr	A_MateM_4d_acc_gland
em18-20hr	A_MateM_4d_head
em20-22hr	A_MateM_4d_testis
em22-24hr	A_1d_carcass
L1	A_1d_dig_sys
L2	A_20d_carcass
L3_12hr	A_20d_dig_sys
L3_PS1-2	A_4d_carcass
L3_PS3-6	A_4d_dig_sys
L3_PS7-9	P8_CNS
WPP	L3_CNS
P5	L3_Wand_carcass
P6	L3_Wand_dig_sys
P8	L3_Wand_fat
P9-10	L3_Wand_imag_disc
P15	L3_Wand_saliv
AdF_Ecl_1days	A_VirF_20d_head
AdF_Ecl_5days	A_VirF_4d_ovary
AdF_Ecl_30days	WPP_fat
AdM_Ecl_1days	WPP_saliv
AdM_Ecl_5days	P8_fat
AdM_Ecl_30days	

Table S2. The 28 genes (27 with five-species orthologs for further follow-up study) that were identified herein as having a high dN/dS value (≥1.5 fold higher than genome-wide median) based on assessment of the gene sets that affected ovariole numbers and/or egg laying in *D. melanogaster* based on RNAi in a *hpo* loss of function genetic background from Kumar, et al. (2020). The observed phenotypes were named as *hpo[RNAi]* Ovariole number, *hpo[RNAi]* Egg laying and Egg laying *[wt]*, or as "connectors". Results are shown for dN/dS obtained for *D. melanogaster-D. simulans* (Dmel-Dsim) (genome-wide median=0.086), the *melanogaster* subgroup (median=0.089), and the *melanogaster* group (0.062) using data available from (Stanley and Kulathinal 2016) and the M0 model of PAML that provides a single dN/dS per alignment (Yang 2007). For completeness, all details for genes that belong to more than one of the four phenotype gene sets described above, and those with dN/dS in more than one of the three sets of *Drosophila* species are shown. Genes are listed in descending order with respect to the M0 dN/dS of the highest value per gene. Occasional genes near the cut-offs were retained for analysis. These genes were subjected to follow-up molecular evolutionary analyses.

Fbgn ID	CG number	Name	Symbol	dN/dS	Species set for dN/dS	Gene phenotype observed/Status
FBgn0011274	CG6794	*Dorsal-related immunity factor*	*Dif*	0.475	*melanogaster* subgroup	*hpo[RNAi]* Egg laying
FBgn0011274	CG6794	*Dorsal-related immunity factor*	*Dif*	0.311	*melanogaster* group	*hpo[RNAi]* Egg laying
FBgn0014020	CG8416	*Rho1*	*Rho1*	0.418	Dmel-Dsim	*hpo[RNAi]* Ovariole number
FBgn0014020	CG8416	*Rho1*	*Rho1*	0.418	Dmel-Dsim	*hpo[RNAi]* Egg laying
FBgn0014020	CG8416	*Rho1*	*Rho1*	0.418	Dmel-Dsim	Egg laying [wt]
FBgn0003612	CG8068	*Suppressor of variegation 2-10*	*Su(var)2-10*	0.387	Dmel-Dsim	*hpo[RNAi]* Ovariole number
FBgn0003612	CG8068	*Suppressor of variegation 2-10*	*Su(var)2-10*	0.387	Dmel-Dsim	*hpo[RNAi]* Egg laying
FBgn0003612	CG8068	*Suppressor of variegation 2-10*	*Su(var)2-10*	0.387	Dmel-Dsim	Egg laying [wt]
FBgn0026379	CG5671	*Phosphatase and tensin homolog*	*Pten*	0.360	Dmel-Dsim	*hpo[RNAi]* Ovariole number
FBgn0026379	CG5671	*Phosphatase and tensin homolog*	*Pten*	0.289	*melanogaster* subgroup	*hpo[RNAi]* Ovariole number
FBgn0026379	CG5671	*Phosphatase and tensin homolog*	*Pten*	0.232	*melanogaster* group	*hpo[RNAi]* Ovariole number
FBgn0000259	CG15224	*Casein kinase II beta subunit*	*CkIIbeta*	0.359	Dmel-Dsim	*hpo[RNAi]* Ovariole number
FBgn0000259	CG15224	*Casein kinase II beta subunit*	*CkIIbeta*	0.359	Dmel-Dsim	*hpo[RNAi]* Egg laying
FBgn0000259	CG15224	*Casein kinase II beta subunit*	*CkIIbeta*	0.359	Dmel-Dsim	Egg laying [wt]
FBgn0000259	CG15224	*Casein kinase II beta subunit*	*CkIIbeta*	0.195	*melanogaster* subgroup	*hpo[RNAi]* Ovariole number
FBgn0000259	CG15224	*Casein kinase II beta subunit*	*CkIIbeta*	0.195	*melanogaster* subgroup	*hpo[RNAi]* Egg laying

FBgn0000259	CG15224	*Casein kinase II beta subunit*	*CkIIbeta*	0.195	*melanogaster* subgroup	Egg laying [wt]
FBgn0035213	CG2199	-	*CG2199*	0.355	Dmel-Dsim	Connector
FBgn0035213	CG2199	-	*CG2199*	0.334	*melanogaster* subgroup	Connector
FBgn0011642	CG32018	*Zyxin*	*Zyx*	0.317	*melanogaster* subgroup	*hpo[RNAi]* Egg laying
FBgn0011642	CG32018	*Zyxin*	*Zyx*	0.290	Dmel-Dsim	*hpo[RNAi]* Egg laying
FBgn0011642	CG32018	*Zyxin*	*Zyx*	0.127	*melanogaster* group	*hpo[RNAi]* Egg laying
FBgn0262614	CG43140	*polychaetoid*	*pyd*	0.316	Dmel-Dsim	*hpo[RNAi]* Ovariole number
FBgn0031610	CG15436	*Parkin Interacting Substrate*	*Paris*	0.290	Dmel-Dsim	Connector
FBgn0036974	CG5605	*eukaryotic translation release factor 1*	*eRF1*	0.267	Dmel-Dsim	*hpo[RNAi]* Ovariole number
FBgn0036974	CG5605	*eukaryotic translation release factor 1*	*eRF1*	0.267	Dmel-Dsim	*hpo[RNAi]* Egg laying
FBgn0036974	CG5605	*eukaryotic translation release factor 1*	*eRF1*	0.267	Dmel-Dsim	Egg laying [wt]
FBgn0003984	CG10491	*vein*	*vn*	0.258	*melanogaster* subgroup	*hpo[RNAi]* Ovariole number
FBgn0003984	CG10491	*vein*	*vn*	0.221	*melanogaster* group	*hpo[RNAi]* Ovariole number
FBgn0004858	CG4220	*elbow B*	*elB*	0.211	*melanogaster* subgroup	*hpo[RNAi]* Ovariole number
FBgn0004858	CG4220	*elbow B*	*elB*	0.138	*melanogaster* group	*hpo[RNAi]* Ovariole number
FBgn0010825	CG6964	*Grunge*	*Gug*	0.211	Dmel-Dsim	*hpo[RNAi]* Ovariole number
FBgn0010825	CG6964	*Grunge*	*Gug*	0.211	Dmel-Dsim	*hpo[RNAi]* Egg laying
FBgn0010825	CG6964	*Grunge*	*Gug*	0.211	Dmel-Dsim	Egg laying [wt]
FBgn0002174	CG5504	-	*CG5504*	0.191	*melanogaster* subgroup	*hpo[RNAi]* Ovariole number
FBgn0002174	CG5504	-	*CG5504*	0.124	*melanogaster* group	*hpo[RNAi]* Ovariole number
FBgn0037218	CG1107	*auxilin*	*aux*	0.190	*melanogaster* subgroup	*hpo[RNAi]* Egg laying

FBgn0037218	CG1107	*auxilin*	*aux*	0.190	*melanogaster* subgroup	Egg laying [wt]
FBgn0037218	CG1107	*auxilin*	*aux*	0.157	Dmel-Dsim	*hpo[RNAi]* Egg laying
FBgn0037218	CG1107	*auxilin*	*aux*	0.157	Dmel-Dsim	Egg laying [wt]
FBgn0037218	CG1107	*auxilin*	*aux*	0.099	*melanogaster* group	*hpo[RNAi]* Egg laying
FBgn0037218	CG1107	*auxilin*	*aux*	0.099	*melanogaster* group	Egg laying [wt]
FBgn0259176	CG42281	*bunched*	*bun*	0.168	*melanogaster* subgroup	*hpo[RNAi]* Ovariole number
FBgn0259176	CG42281	*bunched*	*bun*	0.136	Dmel-Dsim	*hpo[RNAi]* Ovariole number
FBgn0023540	CG3630	-	*CG3630*	0.159	Dmel-Dsim	Connector
FBgn0023540	CG3630	-	*CG3630*	0.138	*melanogaster* subgroup	Connector
FBgn0023540	CG3630	-	*CG3630*	0.098	*melanogaster* group	Connector
FBgn0261854	CG42783	*atypical protein kinase C*	*aPKC*	0.184	*melanogaster* subgroup	*hpo[RNAi]* Egg laying
FBgn0261854	CG42783	*atypical protein kinase C*	*aPKC*	0.098	*melanogaster* group	*hpo[RNAi]* Egg laying
FBgn0001169	CG5460	*Hairless*	*H*	0.155	*melanogaster* subgroup	*hpo[RNAi]* Ovariole number
FBgn0001169	CG5460	*Hairless*	*H*	0.149	Dmel-Dsim	*hpo[RNAi]* Ovariole number
FBgn0001169	CG5460	*Hairless*	*H*	0.148	*melanogaster* group	*hpo[RNAi]* Ovariole number
FBgn0024291	CG5216	*Sirtuin 1*	*Sirt1*	0.153	Dmel-Dsim	*hpo[RNAi]* Egg laying
FBgn0024291	CG5216	*Sirtuin 1*	*Sirt1*	0.153	Dmel-Dsim	Egg laying [wt]
FBgn0030904	CG5988	*unpaired 2*	*upd2*	0.152	*melanogaster* subgroup	*hpo[RNAi]* Ovariole number
FBgn0030904	CG5988	*unpaired 2*	*upd2*	0.152	*melanogaster* subgroup	*hpo[RNAi]* Egg laying
FBgn0030904	CG5988	*unpaired 2*	*upd2*	0.152	*melanogaster* subgroup	Egg laying [wt]
FBgn0030904	CG5988	*unpaired 2*	*upd2*	0.114	*melanogaster* group	*hpo[RNAi]* Ovariole number
FBgn0030904	CG5988	*unpaired 2*	*upd2*	0.114	*melanogaster* group	*hpo[RNAi]* Egg laying
FBgn0030904	CG5988	*unpaired 2*	*upd2*	0.114	*melanogaster* group	Egg laying [wt]
FBgn0020496	CG7583	*C-terminal Binding Protein*	*CtBP*	0.148	Dmel-Dsim	*hpo[RNAi]* Ovariole number

FBgn0020496	CG7583	*C-terminal Binding Protein*	*CtBP*	0.148	Dmel-Dsim	*hpo[RNAi]* Egg laying
FBgn0020496	CG7583	*C-terminal Binding Protein*	*CtBP*	0.148	Dmel-Dsim	Egg laying [wt]
FBgn0003607	CG8409	*Suppressor of variegation 205*	*Su(var)205*	0.144	Dmel-Dsim	Connector
FBgn0261592	CG10944	*Ribosomal protein S6*	*RpS6*	0.139	*melanogaster* subgroup	*hpo[RNAi]* Ovariole number
FBgn0261592	CG10944	*Ribosomal protein S6*	*RpS6*	0.139	*melanogaster* subgroup	*hpo[RNAi]* Egg laying
FBgn0261592	CG10944	*Ribosomal protein S6*	*RpS6*	0.139	*melanogaster* subgroup	Egg laying [wt]
FBgn0020386	CG1210	*Phosphoinositide-dependent kinase 1*	*Pdk1*	0.138	Dmel-Dsim	*hpo[RNAi]* Ovariole number
FBgn0002592	CG6104	*Enhancer of split m2, Bearded family member*	*E(spl)m2-BFM*	0.133	*melanogaster* subgroup	*hpo[RNAi]* Ovariole number
FBgn0002592	CG6104	*Enhancer of split m2, Bearded family member*	*E(spl)m2-BFM*	0.110	*melanogaster* group	*hpo[RNAi]* Ovariole number
FBgn0032006	CG8222	*PDGF- and VEGF-receptor related*	*Pvr*	0.129	*melanogaster* subgroup	*hpo[RNAi]* Egg laying
FBgn0032006	CG8222	*PDGF- and VEGF-receptor related*	*Pvr*	0.117	*melanogaster* group	*hpo[RNAi]* Egg laying
FBgn0045035	CG6535	*telomere fusion*	*tefu*	0.107	*melanogaster* group	*hpo[RNAi]* Ovariole number
FBgn0045035	CG6535	*telomere fusion*	*tefu*	0.107	*melanogaster* group	Egg laying [wt]

Table S3. The 27 rapidly evolving signalling and connector genes under study. For each gene, the associated pathways, *tau* and key functional terms at FlyBase (Gramates, et al. 2017) are shown. From calculation of *tau,* the x̂ for adult virgin ovaries is shown as an example of this parameter (values of 1 underlined). Genes are listed in descending order from the highest M0 dN/dS values from PAML (Table S2). Pathways are from Kumar, et al. (2020)

**Fbgn ID**	**Gene Symbol**	**Pathways**	***tau* 59-All**	**$\hat{x}$ Ovary virgin**	**Key functional terms at FlyBase**
FBgn0011274	*Dif*	Toll	0.717	0.027	Bacterial response
FBgn0014020	*Rho1*	JNK, EGF, Wnt, TGFβ	0.669	0.266	Actin, cytoskeleton
FBgn0003612	*Su(var)2-10*	JAK/STAT	0.786	1.000	Chromosome structure and function
FBgn0026379	*Pten*	mTOR, FOXO	0.663	1.000	Controlling cytoskeletal rearrangements.
FBgn0000259	*CkIIbeta*	Wnt	0.825	0.356	Functions in oogenesis, neurogenesis
FBgn0035213	*CG2199*	Connector	0.808	1.000	Unknown, transcriptional regulation
FBgn0011642	*Zyx*	Hippo	0.647	0.220	Actin cytoskeleton regulator
FBgn0262614	*pyd*	JNK	0.714	0.233	Cytoskeleton
FBgn0036974	*eRF1*	SHH	0.567	0.564	Termination of nascent peptide synthesis
FBgn0003984	*vn*	EGF, Hippo	0.740	0.091	Growth and patterning of tissues
FBgn0004858	*elB*	Notch	0.739	0.042	Development, cell proliferation
FBgn0010825	*Gug*	Hippo	0.706	0.886	Normal developmental patterning
FBgn0002174	*CG5504*	SHH	0.843	0.129	Tumor suppressor, larvae
FBgn0037218	*aux*	Notch	0.674	1.000	Sperm individualization and neuron death
FBgn0259176	*bun*	TGF B	0.606	0.541	Eye development and oogenesis
FBgn0023540	*CG3630*	Connector	0.856	0.097	Actin binding activity, cytoskeleton, Rho activator
FBgn0261854	*aPKC*	Hippo	0.679	0.395	Neuroblast proliferation and self-renewal
FBgn0001169	*H*	Notch	0.829	0.800	Imaginal development
FBgn0024291	*Sirt1*	FOXO	0.671	0.922	Gene silencing, apoptosis, development
FBgn0030904	*upd2*	Hippo, JAK/STAT	0.962	0.000	Development
FBgn0020496	*CtBP*	Wnt, Notch	0.670	0.584	Embryonic segmentation

FBgn0003607	*Su(var)205*	Connector	0.831	0.403	Gene repression, epigenetic repression
FBgn0261592	*RpS6*	mTOR	0.658	0.211	Component of small (40S) ribosomal subunit.
FBgn0020386	*Pdk1*	mTOR, FOXO	0.565	0.857	Embryo development, inhibits apoptosis, spermatogenesis
FBgn0002592	*E(spl)m2-BFM*	Notch	0.871	0.000	Development
FBgn0032006	*Pvr*	VEGF	0.768	0.034	Cell migration regulation
FBgn0045035	*tefu*	FOXO	0.722	0.889	Development, reproduction

Notes: The *Paris* connector gene had *tau*=0.798, $\hat{x}$=1.0 and functions in the negative regulation of transcription.

Table S4. The 27 rapidly evolving SIGNALC genes identified from Kumar, et al. (2020) and their expression status in the soma and germ cells in the larval ovary (each pooled across stages), and among somatic cells at the early, mid and late stages (DeSeq2 P<0.01 (Tarikere, et al. 2022)). Note that if a gene is designated as upregulated in the germ cells, this automatically indicates it is downregulated in soma. A total of 25 of 27 genes showed differential expression using at least one of these comparisons.

**Fbgn ID**	**Gene symbol**	**Upregulation observed** (Tarikere, et al. 2022)	
		Somatic versus germ cells	Somatic cells-stage
FBgn0011274	*Dif*	Soma	Early
FBgn0014020	*Rho1*	Soma	-
FBgn0003612	*Su(var)2-10*	-	Late
FBgn0026379	*Pten*	-	Late
FBgn0000259	*CkIIbeta*	-	Late
FBgn0035213	*CG2199*	-	-
FBgn0011642	*Zyx*	-	Late
FBgn0262614	*Pyd*	Soma	Late
FBgn0036974	*eRF1*	Soma	-
FBgn0003984	*vn*	Soma	-
FBgn0004858	*elB*	Soma	Early
FBgn0010825	*Gug*	Soma	-
FBgn0002174	*CG5504*	-	Early
FBgn0037218	*aux*	-	-
FBgn0259176	*bun*	Soma	-
FBgn0023540	*CG3630*	Germ	-
FBgn0261854	*aPKC*	Soma	-
FBgn0001169	*H*	-	Late
FBgn0024291	*Sirt1*	-	Late
FBgn0030904	*upd2*	Soma	-
FBgn0020496	*CtBP*	-	Late
FBgn0003607	*Su(var)205*	Soma	-
FBgn0261592	*RpS6*	-	Early
FBgn0020386	*Pdk1*	-	Late
FBgn0002592	*E(spl)m2-BFM*	Soma	-
FBgn0032006	*Pvr*	Soma	Late
FBgn0045035	*Tefu*	Germ	-

Notes: the gene *Paris* (FBgn0031610), that was rapidly evolving in Dmel-Dsim but lacked high confidence orthologs in all five species was reported as upregulated in the germ cells relative to the soma cells in (Tarikere, et al. 2022).

Table S5. Genes that were highly upregulated in germ cells of the *D. melanogaster* larval ovary (relative to somatic cells) pooled across three larval stages (Tarikere, et al. 2022) and that exhibited rapid divergence in the *melanogaster* subgroup (M0 dN/dS>0.20). Free-ratio dN/dS per branch, branch-site positive selection and *tau* values are shown for each gene. The genes with the top 10 log_2 fold change values matching these criteria are shown. Upregulation in germ cells implies downregulation in the somatic cells.

**Fbgn ID**	**Log_2 Fold Change**	**Gene name**	**Gene Symbol**	**M0 dN/dS**	**Free-ratio dN/dS**					**Branch-site positive selection P<0.05**					***tau***
					**Dsim**	**Dsec**	**Dmel**	**Dyak**	**Dere**	**Dsim**	**Dsec**	**Dmel**	**Dyak**	**Dere**	
FBgn0031946	27.531	*CG7164*	*CG7164*	0.2652	0.0001	0.2922	0.2698	0.269	0.5347						0.9678
FBgn0031620	10.754	*CG11929*	*CG11929*	0.4818	0.8017	0.3598	0.4851	0.3439	0.5503					yes	0.9407
FBgn0032375	10.728	*CG14932*	*CG14932*	0.6195	>1	0.7898	2.0139	0.2495	0.4939		yes	yes			0.9462
FBgn0047199	10.422	*CG31517*	*CG31517*	1.4448	-	>1	>1	>1	0.5721						0.8702
FBgn0034838	10.363	*CG12782*	*CG12782*	0.2148	0.4158	0.3893	0.1321	0.2894	0.2932						0.9753
FBgn0051475	10.354	*CG31475*	*CG31475*	0.2820	0.1872	0.4101	0.0408	0.1468	0.0676		yes		yes		0.8798
FBgn0034839	10.218	*CG13540*	*CG13540*	0.6728	0.3081	>1	0.7091	102.6503	0.3557				yes		0.9307
FBgn0051619	10.150	*no long nerve cord*	*nolo*	0.2090	0.327	1.0443	0.2099	0.2221	0.1103	yes			yes		0.9218
FBgn0003009	9.910	*orientation disruptor*	*ord*	0.2084	0.0285	0.2861	0.1592	0.2163	0.2876		yes			yes	0.9138
FBgn0039343	9.846	*CG5111*	*CG5111*	0.2013	0.138	0.1722	0.1472	0.1256	0.1282						0.9522

Notes: "-" indicates the dN and dS were each <0.001 and thus too low divergence to determine dN/dS.

Table S6. The genes that were highly upregulated in the somatic cells of the *D. melanogaster* larval ovary in one of three stages of development (early, mid or late) as defined by Tarikere, et al. (2022) and that had elevated M0 dN/dS values in the *melanogaster* subgroup (>0.20). All differentially expressed genes with M0 dN/dS>0.20 were ranked according to $\log_2$ fold change and the 30 genes with the greatest degree of upregulation at one stage of the soma are shown. Also shown is the free ratio branch dN/dS for each species, the presence of branch-site positive selection (indicated by "yes" (P<0.05)), and the *tau* value. "Stage" indicates the developmental stage in which the gene displayed upregulation.

**Fbgn ID**	**$\log_2$ fold change**	**Stage**	**Gene name**	**Symbol**	**M0 dN/dS**	**dN/dS per branch**					**Branch-site positive selection**					***tau***
						**Dsim**	**Dsec**	**Dmel**	**Dyak**	**Dere**	**Dsim**	**Dsec**	**Dmel**	**Dyak**	**Dere**	
FBgn0052057	8.698	Late	*defective proboscis extension response 10*	*dpr10*	0.2104	0.0001	0.6537	0.0983	0.1774	0.1875		yes			yes	0.8784
FBgn0039099	8.607	Late	*Gamma-interferon-inducible lysosomal thiol reductase 2*	*GILT2*	0.2453	0.7806	1.5581	0.0437	0.4597	0.1061						0.9546
FBgn0051296	8.161	Early	*CG31296*	*CG31296*	0.3219	0.3233	0.2084	0.2412	0.3172	0.5849					yes	0.9893
FBgn0015872	7.798	Late	*drip*	*Drip*	0.2734	0.6248	>1	0.4140	0.3750	0.1312	yes		yes	yes		0.9786
FBgn0003651	7.644	Late	*seven up*	*svp*	0.2482	0.0001	0.3980	0.3406	0.0001	0.0001				yes		0.7428
FBgn0085222	7.440	Mid	*CG34193*	*CG34193*	0.2104	0.0001	0.2387	0.4706	0.0001	0.3696						0.9757
FBgn0260943	6.235	Late	*RNA-binding protein 6*	*Rbp6*	0.5015	0.5253	0.0001	0.5512	0.0001	0.0001	yes		yes			0.9251
FBgn0040343	5.951	Late	*CG3713*	*CG3713*	0.2636	>1	0.0001	0.8226	0.1501	0.3225						0.9146
FBgn0033076	5.417	Late	*CG15233*	*CG15233*	0.6252	0.1922	0.6592	1.2160	1.0832	0.7722		yes		yes		0.9498
FBgn0035426	5.001	Late	*CG12078*	*CG12078*	0.2513	0.6469	0.2561	0.2993	0.0911	0.1641						0.9517
FBgn0260479	4.938	Mid	*CG31904*	*CG31904*	0.3038	0.0001	0.4808	0.6401	0.0556	0.1363		yes	yes			0.9466
FBgn0052259	4.863	Late	*CG32259*	*CG32259*	0.9261	>1	1.1050	0.8184	0.5433	1.6762				yes	yes	0.9540
FBgn0026315	4.804	Early	*UDP-glycosyltransferase family 35 member A1*	*Ugt35A1*	0.2279	0.1670	0.2725	0.2604	0.1125	0.3850					yes	0.7528
FBgn0030776	4.620	Mid	*CG4653*	*CG4653*	0.2657	0.1363	0.2587	0.3934	0.1863	0.3557			yes			0.8962
FBgn0032780	4.397	Late	*CG13085*	*CG13085*	0.2262	0.2711	0.4829	0.1077	0.1360	0.3106						0.8017
FBgn0002868	4.380	Early	*Metallothionein A*	*MtnA*	0.6883	-	>1	>1	>1	0.1265						0.8753
FBgn0052246	4.351	Late	*CG32246*	*CG32246*	0.6842	2.9026	1.4628	0.4046	0.3813	0.8883		yes			yes	0.9569
FBgn0033541	4.345	Late	*CG12934*	*CG12934*	0.4946	0.0939	0.4220	0.1443	0.6220	0.7280					yes	0.9633
FBgn0087012	4.305	Early	*5-hydroxytryptamine (serotonin) receptor 2A*	*5-HT2A*	0.3593	0.4309	0.4999	0.3961	0.5155	0.5550					yes	0.8491
FBgn0032780	4.243	Mid	*CG13085*	*CG13085*	0.2262	0.2711	0.4829	0.1077	0.1360	0.3106						0.8017
FBgn0035917	4.230	Late	*Z band alternatively spliced PDZ-motif protein 66*	*Zasp66*	0.2118	0.0001	0.4500	0.0646	0.0340	0.0811						0.8459
FBgn0016075	4.200	Late	*viking*	*vkg*	0.3860	0.3038	0.7557	0.2965	0.3510	0.5572		yes		yes	yes	0.9037
FBgn0004607	4.199	Late	*Zn finger homeodomain 2*	*zfh2*	0.2410	0.1792	0.4571	0.2484	0.2128	0.2496		yes				0.7816
FBgn0039679	4.130	Mid	*pickpocket 19*	*ppk19*	0.2142	0.2244	0.1435	0.3729	0.1705	0.3416						0.9724
FBgn0039938	4.025	Late	*Sox102F*	*Sox102F*	0.2506	0.1676	0.3726	0.2315	0.1600	0.3848						0.7231
FBgn0039679	3.933	Late	*pickpocket 19*	*ppk19*	0.2142	0.2244	0.1435	0.3729	0.1705	0.3416						0.9724
FBgn0000299	3.875	Late	*Collagen type IV alpha 1*	*Col4a1*	0.4065	0.3034	1.2519	0.2072	0.4728	0.6879		yes		yes	yes	0.8887
FBgn0003380	3.700	Early	*Shaker*	*Sh*	0.2416	0.4047	0.0001	0.0001	0.0001	0.0975	yes					0.8968
FBgn0046874	3.519	Late	*PFTAIRE-interacting factor 1B*	*Pif1B*	0.3485	0.4171	0.3360	0.2116	0.3971	0.2675						0.6881
FBgn0030776	3.331	Late	*CG4653*	*CG4653*	0.2657	0.1363	0.2587	0.3934	0.1863	0.3557			yes			0.8962

Notes: "-" indicates the dN and dS were each <0.001 and thus too low divergence to determine dN/dS. "yes" indicates P<0.05 in branch-site selection test.

Table S7. Rapidly evolving genes (M0 dN/dS>0.20 in the *melanogaster* subgroup) that were statistically significantly upregulated in one cell type of the LL3 ovary relative to all other cell types from the Slaidina et al. (2020) sc-RNA seq data (P<0.05 using Seurat program v.2 (where $\log=\log_e$ in v2 (Satija, et al. 2015)). The genes listed include all those with M0 dN/dS>0.20 and with upregulation in the cell type provided. Genes could be upregulated in more than one cell type and thus may be listed more than once (Slaidina, et al. 2020). The subset of genes that were exclusively upregulated in the terminal filament cells (TF) or sheath cells (SH) cells are listed under Notes. The results are shown for the SHa= anterior sheath cells, SHm= migrating sheath cells, TFa= anterior terminal filament cells, TFp= posterior terminal filament cells.

**Fbgn**	**Gene name**	**Symbol**	**Avg_logFC**	**Cell type**	**M0 dN/dS**	**dN/dS per branch**					**Branch-site positive selection**					***tau***
						**Dsim**	**Dsec**	**Dmel**	**Dyak**	**Dere**	**Dsim**	**Dsec**	**Dmel**	**Dyak**	**Dere**	
**Anterior sheath cells (SHa)**																
FBgn0051363	*Jupiter*	*Jupiter*	0.9101	SHa	0.3236	0.0001	0.3714	>1	0.3439	0.2218				yes		0.8004
FBgn0025879	*Tissue inhibitor of metalloproteases*	*Timp*	0.6981	SHa	0.3563	-	0.5309	0.1187	0.152	0.5264		yes				0.7205
FBgn0033438	*Matrix metalloproteinase 2*	*Mmp2*	0.3692	SHa	0.3464	0.0328	0.3251	0.3121	0.2215	0.528					yes	0.8539
FBgn0014133	*bifocal*	*bif*	0.3673	SHa	0.3763	0.2948	0.7923	0.1719	0.1024	0.151		yes				0.7583
FBgn0033724	*CG8501*	*CG8501*	0.3626	SHa	0.4481	>1	0.3405	0.1569	0.782	0.7183	yes					0.8952
FBgn0261552	*pasilla*	*ps*	0.3091	SHa	0.3513	0.1772	1.0481	0.1473	0.1014	0.0519	yes			yes	yes	0.7835
FBgn0260768	*CG42566*	*CG42566*	0.2971	SHa	0.2277	-	0.3063	0.5437	0.0807	0.3225			yes			0.8882
FBgn0000719	*folded gastrulation*	*fog*	0.2724	SHa	0.3551	0.754	0.6134	0.5948	0.4491	0.2274						0.6477
FBgn0003507	*serpent*	*srp*	0.2635	SHa	0.2258	0.3777	0.2928	0.1872	0.1613	0.2537	yes	yes				0.8608
**Migrating sheath cells (SHm)**																
FBgn0025879	*Tissue inhibitor of metalloproteases*	*Timp*	0.9614	SHm	0.3563	-	0.5309	0.1187	0.152	0.5264		yes				0.7205
FBgn0051363	*Jupiter*	*Jupiter*	0.9497	SHm	0.3236	0.0001	0.3714	>1	0.3439	0.2218				yes		0.8004
FBgn0033724	*CG8501*	*CG8501*	0.5135	SHm	0.4481	>1	0.3405	0.1569	0.782	0.7183	yes					0.8952
FBgn0027932	*A kinase anchor protein 200*	*Akap200*	0.4686	SHm	0.3478	0.3369	0.2055	0.5401	0.3198	0.5119						0.8698
FBgn0000299	*Collagen type IV alpha 1*	*Col4a1*	0.4654	SHm	0.4065	0.3034	1.2519	0.2072	0.4728	0.6879		yes		yes	yes	0.8887
FBgn0036101	*Ninjurin A*	*NijA*	0.3684	SHm	0.2904	0.1572	0.4455	0.0821	0.4177	0.1882		yes		yes		0.8700
FBgn0014133	*bifocal*	*bif*	0.3325	SHm	0.3763	0.2948	0.7923	0.1719	0.1024	0.151		yes				0.7583
FBgn0261552	*pasilla*	*ps*	0.3178	SHm	0.3513	0.1772	1.0481	0.1473	0.1014	0.0519	yes			yes	yes	0.7835
FBgn0016075	*viking*	*vkg*	0.3121	SHm	0.3860	0.3038	0.7557	0.2965	0.351	0.5572		yes		yes	yes	0.9037
FBgn0033438	*Matrix metalloproteinase 2*	*Mmp2*	0.3011	SHm	0.3464	0.0328	0.3251	0.3121	0.2215	0.528					yes	0.8539
FBgn0051955	*CG31955*	*CG31955*	0.2604	SHm	0.6646	5.8793	0.2682	0.7278	0.5471	0.7476					yes	0.8951
**Anterior terminal filament cells (TFa)**																
FBgn0015229	*gliolectin*	*glec*	1.7677	TFa	0.5567	0.14	0.1492	0.8271	0.4095	0.6083						0.6855
FBgn0034199	*Growth-blocking peptide 1*	*Gbp1*	1.6379	TFa	0.4162	>1	0.4375	0.0001	1.1754	0.3524				yes		0.9484
FBgn0025879	*Tissue inhibitor of metalloproteases*	*Timp*	1.4819	TFa	0.3563	-	0.5309	0.1187	0.152	0.5264		yes				0.7205
FBgn0034198	*CG11400*	*CG11400*	1.4270	TFa	0.3592	0.0001	0.188	0.3328	1.3207	0.355						0.7854
FBgn0001253	*Ecdysone-inducible gene E1*	*ImpE1*	1.3992	TFa	0.2267	0.2315	0.5900	0.2456	0.1932	0.2401		yes				0.9160
FBgn0034638	*CG10433*	*CG10433*	1.0711	TFa	0.2948	0.0001	0.8546	0.0001	0.1363	0.0362						0.8591
FBgn0262563	*CG43103*	*CG43103*	1.0423	TFa	0.2694	0.0001	0.3256	0.1094	0.4067	0.4623				yes		0.7215
FBgn0033134	*Tetraspanin 42El*	*Tsp42El*	0.9933	TFa	0.2052	0.0001	0.133	0.1603	0.1166	0.39						0.7592
FBgn0001227	*Heat shock gene 67Ba*	*Hsp67Ba*	0.9834	TFa	0.2828	0.0001	0.7352	0.1914	0.2109	0.4008		yes			yes	0.9249

FBgn0261822	*Basigin*	*Bsg*	0.9380	TFa	0.3411	0.1543	0.4645	0.0416	0.7968	0.157		yes		yes		0.6604
FBgn0038071	*Dpp target gene*	*Dtg*	0.8901	TFa	0.3946	0.2878	0.3957	0.503	0.4808	0.506						0.8793
FBgn0036101	*Ninjurin A*	*NijA*	0.8596	TFa	0.2904	0.1572	0.4455	0.0821	0.4177	0.1882		yes		yes		0.8700
FBgn0020503	*Cytoplasmic linker protein 190*	*CLIP-190*	0.7221	TFa	0.2379	0.1466	0.4152	0.2074	0.2021	0.1592		yes				0.6998
FBgn0015872	*Drip*	*Drip*	0.6456	TFa	0.2734	0.6248	>1	0.414	0.375	0.1312	yes		yes	yes		0.9786
FBgn0003435	*smooth*	*sm*	0.6382	TFa	0.3992	>1	0.6092	0.0309	0.0001	0.5679		yes				0.8599
FBgn0031968	*CG7231*	*CG7231*	0.6366	TFa	0.2445	0.3018	0.6806	0.1559	0.1826	0.3635		yes				0.8268
FBgn0034398	*CG15098*	*CG15098*	0.5951	TFa	0.4849	>6	0.743	0.8262	0.3037	0.6975					yes	0.7146
FBgn0032949	*Lamp1*	*Lamp1*	0.5880	TFa	0.3451	0.0001	0.4433	0.5723	0.3839	0.4147						0.8346
FBgn0038476	*kugelkern*	*kuk*	0.5606	TFa	0.2970	0.1612	0.3325	0.3852	0.1806	0.3661						0.9247
FBgn0000299	*Collagen type IV alpha 1*	*Col4a1*	0.5519	TFa	0.4065	0.3034	1.2519	0.2072	0.4728	0.6879		yes		yes	yes	0.8887
FBgn0033889	*CG6701*	*CG6701*	0.5396	TFa	0.3091	0.245	1.0488	0.3838	0.288	0.4045		yes				0.6936
FBgn0030174	*CG15312*	*CG15312*	0.5228	TFa	0.3686	0.0001	0.6218	0.1324	0.5161	0.1388		yes				0.8095
FBgn0038682	*CG5835*	*CG5835*	0.4978	TFa	0.3324	0.0384	0.1801	0.6696	0.2774	0.5286						0.8715
FBgn0030237	*CG15209*	*CG15209*	0.4724	TFa	0.2491	0.2992	0.2840	0.2506	0.1882	1.1764						0.7312
FBgn0051361	*defective proboscis extension response 17*	*dpr17*	0.4614	TFa	0.3000	0.2126	0.2577	0.255	0.6819	0.3233						0.8514
FBgn0035020	*CG13585*	*CG13585*	0.4568	TFa	0.2637	0.2087	>1	0.1471	0.0931	0.188						0.9061
FBgn0086686	*lethal (3) L1231*	*l(3)L1231*	0.4452	TFa	0.2198	0.1294	0.4058	0.2666	0.1919	0.2303						0.6575
FBgn0040343	*CG3713*	*CG3713*	0.4337	TFa	0.2636	>1	0.0001	0.8226	0.1501	0.3225						0.9146
FBgn0085407	*PDGF- and VEGF-related factor 3*	*Pvf3*	0.4201	TFa	0.2440	0.0245	0.3884	0.0525	0.1055	0.1217						0.9222
FBgn0016075	*viking*	*vkg*	0.4074	TFa	0.3860	0.3038	0.7557	0.2965	0.351	0.5572		yes		yes	yes	0.9037
FBgn0039914	*maverick*	*mav*	0.3770	TFa	0.2504	0.3964	0.532	0.5972	0.2124	0.4124						0.6731
FBgn0038498	*beaten path IIa*	*beat-IIa*	0.3759	TFa	0.2430	0.1713	0.0001	0.2514	0.3582	0.2106						0.8736
FBgn0034200	*Growth-blocking peptide 2*	*Gbp2*	0.3751	TFa	0.3622	0.3128	0.2299	0.3764	0.294	0.3957						0.8323
FBgn0027598	*CIN85 and CD2AP related*	*cindr*	0.3680	TFa	0.3297	0.2759	0.6744	0.0758	0.091	0.0772	yes	yes				0.6435
FBgn0035452	*CG10359*	*CG10359*	0.3640	TFa	0.2203	0.2327	0.132	0.3775	0.2047	0.3199						0.8757
FBgn0085432	*pangolin*	*pan*	0.3497	TFa	0.3218	0.2243	0.5643	0.1367	0.126	0.165	yes					0.8586
FBgn0010415	*Syndecan*	*Sdc*	0.3348	TFa	0.3006	0.6124	0.6265	0.2136	0.2055	0.5697		yes			yes	0.8616
FBgn0045842	*yuri gagarin*	*yuri*	0.3305	TFa	0.3037	0.2003	0.3102	0.4189	0.255	0.2499						0.9364
FBgn0037350	*Diphthamide biosynthesis 4*	*CG2911*	0.3238	TFa	0.2303	0.0001	0.4093	0.5159	0.2446	0.1918				yes		0.9143
FBgn0259740	*CG42394*	*CG42394*	0.3003	TFa	0.3499	0.0001	0.0001	2.6837	0.0001	0.2091						0.7401
FBgn0052803	*Lipid droplet assembly factor 1*	*CG32803*	0.2780	TFa	0.2664	0.0001	0.4282	0.8583	0.311	0.1959			yes			0.6557
FBgn0033683	*CG18343*	*CG18343*	0.2762	TFa	1.0011	1.2209	0.4346	4.108	0.3598	1.0348			yes	yes		0.5615
FBgn0026620	*transforming acidic coiled-coil protein*	*tacc*	0.2659	TFa	0.3878	0.2304	0.5105	0.3432	0.4609	0.435		yes			yes	0.5372
FBgn0034724	*babos*	*babos*	0.2632	TFa	0.2450	0.7171	0.0834	0.2045	0.2332	0.3787						0.7684
**Posterior terminal filament cells (TFp)**																
FBgn0034199	*Growth-blocking peptide 1*	*Gbp1*	1.6762	TFp	0.4162	>1	0.4375	0.0001	1.1754	0.3524				yes		0.9484
FBgn0000299	*Collagen type IV alpha 1*	*Col4a1*	1.4987	TFp	0.4065	0.3034	1.2519	0.2072	0.4728	0.6879		yes		yes	yes	0.8887
FBgn0015229	*gliolectin*	*glec*	1.4901	TFp	0.5567	0.14	0.1492	0.8271	0.4095	0.6083						0.6855
FBgn0001253	*Ecdysone-inducible gene E1*	*ImpE1*	1.3009	TFp	0.2267	0.2315	0.5900	0.2456	0.1932	0.2401		yes				0.9160

FBgn0025879	*Tissue inhibitor of metalloproteases*	*Timp*	1.1705	TFp	0.3563	-	0.5309	0.1187	0.152	0.5264		yes				0.7205
FBgn0262563	*CG43103*	*CG43103*	1.1400	TFp	0.2694	0.0001	0.3256	0.1094	0.4067	0.4623				yes		0.7215
FBgn0002868	*Metallothionein A*	*MtnA*	1.0706	TFp	0.6883	-	>1	>1	>1	0.1265						0.8753
FBgn0033134	*Tetraspanin 42El*	*Tsp42El*	0.9692	TFp	0.2052	0.0001	0.133	0.1603	0.1166	0.39						0.7592
FBgn0016075	*viking*	*vkg*	0.9189	TFp	0.3860	0.3038	0.7557	0.2965	0.351	0.5572		yes		yes	yes	0.9037
FBgn0034198	*CG11400*	*CG11400*	0.8837	TFp	0.3592	0.0001	0.188	0.3328	1.3207	0.355						0.7854
FBgn0001227	*Heat shock gene 67Ba*	*Hsp67Ba*	0.8687	TFp	0.2828	0.0001	0.7352	0.1914	0.2109	0.4008		yes			yes	0.9249
FBgn0034638	*CG10433*	*CG10433*	0.7609	TFp	0.2948	0.0001	0.8546	0.0001	0.1363	0.0362						0.8591
FBgn0015872	*Drip*	*Drip*	0.7607	TFp	0.2734	0.6248	>1	0.414	0.375	0.1312	yes		yes	yes		0.9786
FBgn0036101	*Ninjurin A*	*NijA*	0.7144	TFp	0.2904	0.1572	0.4455	0.0821	0.4177	0.1882		yes		yes		0.8700
FBgn0259740	*CG42394*	*CG42394*	0.6697	TFp	0.3499	0.0001	0.0001	2.6837	0.0001	0.2091						0.7401
FBgn0031968	*CG7231*	*CG7231*	0.5292	TFp	0.2445	0.3018	0.6806	0.1559	0.1826	0.3635		yes				0.8268
FBgn0035020	*CG13585*	*CG13585*	0.4914	TFp	0.2637	0.2087	>1	0.1471	0.0931	0.188						0.9061
FBgn0052803	*Lipid droplet assembly factor 1*	*CG32803*	0.4869	TFp	0.2664	0.0001	0.4282	0.8583	0.311	0.1959			yes			0.6557
FBgn0003435	*smooth*	*sm*	0.4832	TFp	0.3992	>1	0.6092	0.0309	0.0001	0.5679		yes				0.8599
FBgn0038476	*kugelkern*	*kuk*	0.4056	TFp	0.2970	0.1612	0.3325	0.3852	0.1806	0.3661						0.9247
FBgn0010415	*Syndecan*	*Sdc*	0.4054	TFp	0.3006	0.6124	0.6265	0.2136	0.2055	0.5697		yes			yes	0.8616
FBgn0261822	*Basigin*	*Bsg*	0.3741	TFp	0.3411	0.1543	0.4645	0.0416	0.7968	0.157		yes		yes		0.6604
FBgn0033683	*CG18343*	*CG18343*	0.3315	TFp	1.0011	1.2209	0.4346	4.108	0.3598	1.0348			yes	yes		0.5615
FBgn0034200	*Growth-blocking peptide 2*	*Gbp2*	0.2992	TFp	0.3622	0.3128	0.2299	0.3764	0.294	0.3957						0.8323
FBgn0038498	*beaten path IIa*	*beat-IIa*	0.2919	TFp	0.2430	0.1713	0.0001	0.2514	0.3582	0.2106						0.8736
FBgn0035452	*CG10359*	*CG10359*	0.2855	TFp	0.2203	0.2327	0.132	0.3775	0.2047	0.3199						0.8757
FBgn0034398	*CG15098*	*CG15098*	0.2681	TFp	0.4849	>1	0.743	0.8262	0.3037	0.6975					yes	0.7146
FBgn0030174	*CG15312*	*CG15312*	0.2649	TFp	0.3686	0.0001	0.6218	0.1324	0.5161	0.1388		yes				0.8095
FBgn0038071	*Dpp target gene*	*Dtg*	0.2564	TFp	0.3946	0.2878	0.3957	0.503	0.4808	0.506						0.8793
FBgn0043841	*virus-induced RNA 1*	*vir-1*	0.2541	TFp	0.2419	0.1295	0.111	0.1677	0.4729	0.3974				yes		0.7401

Notes: Genes that were exclusively upregulated in the SH cells: FBgn0051363, FBgn0033438, FBgn0014133, FBgn0033724, FBgn0000719, FBgn0003507, FBgn0027932, FBgn0051955. Genes that were exclusively upregulated in the TFs: FBgn0001253, FBgn0003435, FBgn0015872, FBgn0020503, FBgn0026620, FBgn0027598, FBgn0030174, FBgn0030237, FBgn0033134, FBgn0033683, FBgn0034198, FBgn0034200, FBgn0034638, FBgn0037350, FBgn0038071, FBgn0038476, FBgn0038498, FBgn0038682, FBgn0039914, FBgn0040343, FBgn0045842, FBgn0051361, FBgn0085407,FBgn0085432, FBgn0086686, FBgn0262563. Branch-site positive selection in *D. simulans*, *D. sechellia* and *D. melanogaster* using the subset of genes exclusively upregulated in SH cells was 25.0, 25.0 and 0% of studied genes respectively and for TFs was 11.5, 23.1, and 7.7% respectively. "-" indicates the dN and dS were each <0.001 and thus too low divergence to determine dN/dS.

Table S8. The top GO functional classifications of rapidly evolving genes that were upregulated in TFa and TFp cell types. Classifications were determined using DAVID (Huang da, et al. 2009) for all genes per category. P-values indicate the statistical significance of preferential classification of the gene into each GO group. Genes could match more than one GO group.

**Functional class**	**Percent of genes matching**	**P-value**
**Terminal filament cells anterior (a)**		
basement membrane organization	6.8	9.10E-04
cytokine activity	6.8	1.00E-03
extracellular space	20.5	2.80E-03
cell adhesion	6.8	1.50E-02
growth factor activity	4.5	4.30E-02
extracellular matrix structural constituent	4.5	5.80E-02
**Terminal filament cells posterior (p)**		
basement membrane organization	10	3.70E-04
extracellular space	23.3	4.10E-03
cell adhesion	10	6.50E-03
collagen type IV trimer	6.7	6.80E-03
cytokine activity	6.7	2.90E-02
extracellular matrix structural constituent	6.7	3.60E-02
integral component of membrane	40	3.80E-02
axon guidance	10	3.90E-02
extracellular matrix	10	4.20E-02
	6.7	4.40E-02

Table S9. The results for the McDonald and Kreitman (1991)tests of positive selection for the 42 genes identified in Tables 1, 2, and 3. Only those genes with P<0.05 are shown. The *D. melanogaster* populations examined are the Raleigh North Carolina (RAL) and the Zambia population. The P-values are shown as MK-P. Alpha (α) indicates the proportion of substitutions apt to have experienced positive selection. Analysis was conducted using the integrative McDonald Kreitman test (Murga-Moreno, et al. 2019) that uses *D. simulans* as the comparison species (Dmel-Dsim). Note that iMKT may not necessarily have the exact same alignment per gene as FlyDivas (Stanley and Kulathinal 2016), which was used for all our core analyses, but the alignments from both analyses are highly similar and representative per gene for this supplementary assessment. Data are available publicly for each dataset (Stanley and Kulathinal 2016; Murga-Moreno, et al. 2019). MK-P indicates the P-value.

		**RAL population**		**Zambia population**	
Fbg ID	Gene	α	MK-P	α	MK-P
FBgn0026379	*Pten*	0.880	0,012	0.771	0.030
FBgn0004858	*elB*	0.756	0.057	0.703	0.001
FBgn0010825	*Gug*	0.530	0.010	0.598	0
FBgn0261854	*aPKC*	0.969	0	0.944	0
FBgn0032006	*Pvr*	0.558	0.020	0.525	0.014
FBgn0039108	*CG10232*	0.632	0.031	0.669	0.002
FBgn0016075	*vkg*	0.681	0	0.769	0
FBgn0000299	*Col4a1*	0.711	0,001	0,757	0

Table S10. The branch dN/dS and branch-site analysis Hawaiian *Drosophila* (*D. murphyi*, *D. sproati* and *D. grimshawi*) for orthologs to the SIGNALC genes identified in table 1 that exhibited signs of involvement in ovariole number evolution (from analysis of the *melanogaster* subgroup, and (Kumar, et al. 2020)). Genes having a dN/dS value of >1 at the gene-wide level and those with values above 0.330 in at least one species branch are each shown (that is double the median of the genome with the highest median dN/dS, *D. sproati*). Species that exhibited positive selection using branch-site (BR-S) analysis in PAML (P<0.05) are indicated by the abbreviated species name.

Fbgn	Gene name	Symbol	Hawaiian branch-dN/dS			BR-S positive selection		
			D. murphyi	*D. sproati*	*D. grimshawi*			
**Genes with dN/dS>0.330 in at least one branch**								
FBgn0030904	*unpaired 2*	*upd2*	**2.7852**	1.6904	1.1397	Dmur	Dspr	Dgri
FBgn0035213	*CG2199*	*CG2199*	**0.637**	0.5455	0.5134			
FBgn0003984	*vein*	*vn*	0.1162	**0.3489**	**0.3484**			Dgri
FBgn0004858	*elbow B*	*elB*	0.0738	**0.4486**	0.0744		Dspr	
FBgn0259176	*bunched*	*bun*	0.5683	0.3779	**0.7306**	Dmur		Dgri
FBgn0023540	*CG3630*	*CG3630*	**0.5018**	0.2986	0.3435			
FBgn0261854	*atypical protein kinase C*	*aPKC*	0.1756	0	**0.5173**			Dgri
FBgn0001169	*Hairless*	*H*	0.3588	**0.8827**	0.4621			Dgri
FBgn0003607	*Suppressor of variegation 205*	*Su(var)205*	0.4541	**0.4620**	0.1688			
FBgn0002592	*Enhancer of split m2, Bearded family member*	*E(spl)m2-BFM*	NA	**0.3478**	0.2298			
**Genes with all branches dN/dS≤0.330**								
FBgn0014020	*Rho1*	*Rho1*	NA	NA	0			
FBgn0003612	*Suppressor of variegation 2-10*	*Su(var)2-10*	0.0001	0.0001	0.2512			Dgri
FBgn0000259	*Casein kinase II beta subunit*	*CkIIbeta*	0.0001	NA	0.0001			
FBgn0262614	*polychaetoid*	*pyd*	0.191	0.0652	0.0777	Dmur		
FBgn0036974	*eukaryotic translation release factor 1*	*eRF1*	NA	0	0.0001			
FBgn0002174	*CG5504*	*CG5504*	0.1637	0.1607	0.2089			Dgri
FBgn0037218	*auxilin*	*aux*	0.2491	0.1765	0.1276			

FBgn0024291	*Sirtuin 1*	*Sirt1*	0.2621	0.294	0.2123
FBgn0020496	*C-terminal Binding Protein*	*CtBP*	0	0.0001	0.0001
FBgn0261592	*Ribosomal protein S6*	*RpS6*	0.0001	0.0001	0.0274
FBgn0045035	*telomere fusion*	*tefu*	0.2727	0.1711	0.2456
**No Orthologs**					
FBgn0011274	*Dorsal-related immunity factor*	*Dif*	No Orth-HD		
FBgn0026379	*Phosphatase and tensin homolog*	*Pten*	No Orth-HD		
FBgn0011642	*Zyxin*	*Zyx*	No Orth-HD		
FBgn0010825	*Grunge*	*Gug*	No Orth-HD		
FBgn0020386	*Phosphoinositide-dependent kinase 1*	*Pdk1*	No Orth-HD		
FBgn0032006	*PDGF- and VEGF-receptor related*	*Pvr*	No Orth-HD		

Notes: No Orth-HD=no high confidence reciprocal BLASTX orthologs identified among the three Hawaiian *Drosophila* (HD). NA indicates the branch did not meet the criteria that dN or dS>0.001 for dN/dS analysis. Ten genes had dN/dS>2 fold higher (value>0.330) than the genome-wide medians in at least one species branch (the branch with the highest dN/dS value is in bold). Three additional genes, *aux*, *Sirt1*, and *tefu* had an elevated value when using a criterion of dN/dS >1.5 fold higher (value>0.246) than the genome-wide medians.

SUPPLEMENTARY TEXT FILE S1

Supplementary Methods for the *melanogaster* subgroup

Identification of Rapidly Evolving Ovariole-Related Genes

With respect to the SIGNALC dataset (Kumar, et al. 2020), for each of four gene sets identified therein as having roles in regulating egg-laying and/or ovariole numbers, namely *hpo[RNAi]* Ovariole Number, *hpo[RNAi]* Egg Laying, Egg Laying [*wt*], and the so-called "Connectors" (genes not included in the initial screen but identified as candidates by network topology analysis), we identified the subset of genes that had orthologs in all five species of the *melanogaster* subgroup and exhibited a markedly elevated M0 dN/dS value relative to the genome-wide values (Yang 2007) using data from FlyDivas (Stanley and Kulathinal 2016). Given that signalling genes are typically components of complex pathways and have pleiotropic roles in a wide range of cells or tissues (Cui, et al. 2009; Kumar, et al. 2020), they may be expected to typically, but, not always (Cui, et al. 2009; Darfour-Oduro, et al. 2016), have been subjected to strong purifying selection and thus evolve slowly in their protein sequence (Cui, et al. 2009; Mank and Ellegren 2009; Meisel 2011; Assis, et al. 2012; Darfour-Oduro, et al. 2016; Masalia, et al. 2017; Whittle, et al. 2021). We therefore defined rapidly evolving ovariole-related genes as those having a value of ≥1.5 fold above the genome-wide median M0 dN/dS in the *melanogaster* subgroup (genome-wide median M0 dN/dS=0.0887, N=9,232). The cut-off was 1.5 (M0 dN/dS value ≥0.133) rather than higher, as for the other studied datasets (see below, BULKSG and SINGLEC), given the innate conserved nature of these genes. In addition, for this one dataset, the SIGNALC, to identify the widest scope possible of genes with a propensity for rapid evolution for follow-up study, we also included any genes with ≥1.5 fold higher M0 dN/dS in the *D. simulans*-*D. melanogaster* pair (≥1.5 fold higher than the median=0.857, N=10,765 aligned genes) and the six-species *melanogaster* group (which includes *D. ananassae* as a outgroup, fig. 2; genome-wide median=0.062, N=8,696 genes) (Stanley and Kulathinal 2016). These genes were added to the gene list for our follow-up study within the *melanogaster* subgroup (fig. 2).

For the BULKSG transcription dataset (Tarikere, et al. 2022), we identified and extracted all genes that were statistically significantly upregulated (using DeSeq2 $P<0.01$ criterion in that assessment (Love, et al. 2014)) in the *D. melanogaster* pooled larval ovary somatic cells and those upregulated in the pooled germ cells. Upregulated in germ cells automatically indicates downregulation in soma cells, and thus both up- and downregulated genes in soma were studied. We also extracted for study those genes that were upregulated at a single one of the larval stages studied (early, mid, or late) for the pooled ovary somatic cells; these stages correspond to different stages of TF formation (Tarikere, et al. 2022). For these gene sets, we then identified those genes with M0 dN/dS>0.20 in the *melanogaster* subgroup, which represent a value ≥2.2 fold higher than the genome-wide median. The cut-off was higher for the BULKSG than SIGNALC dataset, given that we examined all genes in the genome that were differentially expressed in the former dataset, which are apt to be less conserved as a group than the SIGNALC genes. The genes matching these criteria (dN/dS>0.20, and up- or downregulated in soma cells), were ranked based on degree of differential expression, and subjected to follow-up analysis in the *melanogaster* subgroup.

With respect to the SINGLEC dataset (Slaidina, et al. 2020), genes were extracted for further study that were statistically significantly differentially transcribed in a specific cell type of the LL3 larval ovary relative to all other cell types based on sc-RNA seq (using average standardized expression and P-values from Seurat v.2; some genes were upregulated in more than one cell type based on these criteria (Slaidina, et al. 2020)). The nine cell types studied (shown in fig. 1) included the germ cells (GC) and eight somatic cell types, namely the cap cells (CC), follicle stem cell precursors (FSCP), intermingled cells (IC), anterior sheath cells (SHa), migrating sheath cells (SHm), swarm cells (SW), anterior terminal filaments (TFa), and posterior terminal filaments (TFp), with a particular focus on the two types of SH cells and the two types of TF cells, which are thought to largely shape species-specific ovariole numbers and function (King, et al. 1968; Sarikaya, et al. 2012; Sarikaya and Extavour 2015; Slaidina, et al. 2020). Similar to the BULKSG assessment, among the genes that were upregulated per cell type, we identified the genes that had signs of rapid protein sequence divergence (M0 dN/dS >0.20 in genes with five-species orthologs) in the *melanogaster* subgroup (Stanley and Kulathinal 2016; Gramates, et al. 2017), for further analyses.

PGLS Analysis

Any species branch for a gene with an infinity value for dN/dS (denoted as "dN/dS>1" and cited in the tables; that is, dN>0.001, and dS near or at 0); table 1, table 2, table3) was assigned a value of 1.5 for PGLS analysis to indicate that the value was larger than one, while conservatively ascribing a low range value (between one and infinity). If more than one branch had "dN/dS>1", then the gene was excluded from the PGLS (one case, FBgn0002868 in table 3). Any species branch for a gene without a dN/dS value (dN and dS<0.001) was assigned "N/A" for PGLS.

***tau* Analysis**

The *tau* value is a relative measure, and can vary with the number of tissues/stages studied. For instance, we found that *tau* values across all studied genes in the genome determined using all 59 tissues/stages yielded elevated values as compared to using only the 30 developmental stages alone (median= 0.830 versus 0.727, fig. S2), and the two sets of values were strongly correlated (R=0.873, $P<10^{-6}$) suggesting that both values effectively reflect *tau*. Throughout this study, we used the complete available dataset of 59 tissues/stages for all *tau* analyses, and considered genes with values >0.90 as narrowly transcribed (just below the 75th percentile), while those with values below 0.73 (at the 25th percentile) were considered as broadly transcribed, with all other values considered relative to each other and intermediate in *tau*.

Supplementary Methods for the Three-Species Hawaiian Clade

As a supplementary analyses we studied a three species clade of Hawaiian *Drosophila*, shown in fig. S5 (phylogeny from (Kim, et al. 2021; Suvorov, et al. 2022)), that included *D. sproati* (Dspr, N=65.6 ovarioles), *D. murphyi* (Dmur, N=41.6 ovarioles) and *D. grimshawi* (47.8 ovarioles) (ovariole numbers from (Starmer, et al. 2003; Sarikaya, et al. 2019). For *D. sproati* and *D. murphyi* and the genomes were available as unannotated scaffolds from the SRA (Starmer, et al. 2003; Suvorov, et al. 2022) and the closely related species *D. grimshawi* was used as a reference (that has an annotated genome, N=12,825 CDS; longest CDS per gene; all three species genome data under project PRJNA675888 at the SRA database). Using the program Maker2 (Holt and Yandell 2011) and the RNA and proteins sequence list for *D. grimshawi*, we searched *D. sproati*

and *D. murphyi* scaffolds for evidence-based gene predictions. We also obtained ab initio gene predictions in Augustus (Hoff and Stanke 2013) which recommends *D. melanogaster* gene models as a reference for predictions in Dipteran species (Stanke and Waack 2003). In the assessments, we excluded any CDS that lacked a start or a stop codon, or had an internal stop codon, such that only complete CDS were used for study. Using these criteria, we identified 13,395 CDS for *D. murphyi* and 12,670 CDS for *D. sproati* . Thus, these comprise high quality full-length CDS. To assess the completeness of our final *D. sproati* and *D. murphyi* CDS lists we applied BUSCO v. 5.5.2 (Seppey, et al. 2019; Manni, et al. 2021), a program that evaluates the completeness of a given transcriptome by comparing it to a subset of "universal" single-copy genes. For this, we used as the control the Dipteran gene set, named diptera_odb10 (N genes=3,250). The results showed that for the 13,395 full-length CDS for *D. murphyi*, 93.4% had a complete (unfragmented) match in the BUSCO dataset, indicating we obtained a very complete CDS list for this species (note that this value would be elevated using all 14,108 CDS identified for *D. murphyi*, but we focused solely on the obtained full-length CDS). In turn, for *D. sproati* , we found 93.2% of the full-length CDS were represented in the Dipteran BUSCO dataset. Taken together, the results show that our approach yielded a high confidence and highly complete CDS list for *D. sproati* and *D. murphyi.*

We next used reciprocal BLASTX (BLAST v. 2.13.0+ (Altschul, et al. 1997)) to identify orthologs between all three Hawaiian species using all CDS per genome in each contrast, for each combination of paired species, namely *D. grimshawi*-*D. murphyi*, *D. grimshawi*-*D. sproati* and *D. murphyi*-*D. sproati*. For each pair, in order to be identified as orthologs, two CDS had to be the best match (lowest e-score, any matched with a tied e-score the best match was the one with the highest bit value, $e<10^{-6}$) in both the forward and reverse contrast and have an e-value $<10^{-6}$. Using the three sets of paired orthologs, we then compared the orthologs identified in the newly annotated *D. murphyi*-*D. sproati* pair to the *D. grimshawi*.-*D. murphyi* and *D. grimshawi*. -*D. murphyi* pairs, to ensure that the orthologs per gene identified between *D. murphyi* and *D. sproati* each independently matched the same single CDS in *D. grimshawi*. Thus, this comprises very stringent criteria for identification of three-way reciprocal orthologs among *D. grimshawi*.-*D. murphyi*-*D. sproati*.

To identify Hawaiian species orthologs to the genes linked to ovariole/egg number evolution obtained from analysis within the *melanogaster* subgroup (SIGNALC in table 1;

SIGNALC in fig. 4), and for genome-wide orthologs, we compared the CDS of the most well annotated *melanogaster* species *D. melanogaster* (13,986 coding genes with longest isoform per gene) to the annotated Hawaiian species *D. grimshawi* CDS lists using reciprocal BLASTX (using same criteria described for Hawaiian *Drosophila* above). We report that 11,011 high confidence orthologs were identified between *D. melanogaster* and *D. grimshawi* (78.7% of the Dmel CDS list). A total of 10,276 of those genes had high confidence three-species orthologs in Hawaiian clade and were used for study of dN/dS and ortholog detection rates.

Each set of three-species orthologs among *D. grimshawi*.-*D. murphyi*-*D. sproati* were aligned using MUSCLE (Edgar 2004) set to default parameters in MEGA (Kumar, et al. 2016). Each alignment was filtered in GBlocks v. 0.91b set at default parameters (Castresana 2000; Talavera and Castresana 2007) to remove gaps and any highly divergent segments. For each aligned CDS in *D. grimshawi*, *D. murphyi* and *D. sproati*(gaps removed), we determined dN/dS per species branch using the free-ratios model of PAML (M1) and conducted branch-site analyses (Yang 2007), as outlined for the *melanogaster* subgroup in the main text. Branches were unsaturated in substitutions, and the 90th percentile of dS values across all genes was <0.090 and of dN was <0.032 for each of the three Hawaiian species branches. The median gene-wide dN/dS value across all genes for each species branch were 0.152, 0.164, and 0.160 for *D. murphyi*, *D. sproati* and *D. grimshawi* respectively.

Supplementary Results for the *Drosophila melanogaster* subgroup

Descriptions of Genes Linked to Ovariole Evolution

Each of the ovariole-related genes listed in the tables herein has properties suggesting a role in interspecies ovariole number divergence. In the following sections, we highlight some of these genes, which we consider especially promising candidates for functional analyses in future work, and briefly describe published evidence for their role in ovariole number determination and/or interspecies ovariole number divergence. We note that none of the 42 ovariole-related genes identified herein as linked to interspecies divergence in ovariole numbers (tables 1-5, fig. 4) overlapped with any of the 24 genes identified using a recent genome-wide association assessment of variation in 205 lines of the *D. melanogaster* Raleigh population (Lobell, et al. 2017). This is unsurprising, given the latter study was focused on intraspecies variation and ovariole numbers in a single *D. melanogaster* population (unfixed variation), while the present study focused on fixed nonsynonymous (to synonymous, dN/dS) and adaptive changes at the interspecies level

SIGNALC genes (table 1)

<u>*unpaired 2 (unp2)*</u>

The gene *upd2* is a ligand for the JAK/STAT pathway, which interacts with the Hippo pathway during ovariole development (Sarikaya and Extavour 2015). Of the five species in the *melanogaster* subgroup, *upd2* exhibited the highest terminal branch dN/dS value in *D. sechellia* (value of 0.417; table 1) and had signals of branch-site positive selection ($P<0.05$) in this same lineage (and in *D. erecta*, table 1). Thus, the gene protein changes in *D. sechellia* were coupled with the fact that this species has the lowest ovariole number of the species examined (fig. 2). *unp2* also exhibited the highest *tau* value (0.962) of all 27 studied SIGNALC genes, indicating that this gene is narrowly transcribed, which could potentially provide flexibility to adaptively evolve new functions in the ovarioles (table 1; (Otto 2004; Larracuente, et al. 2008; Mank and Ellegren 2009; Whittle, et al. 2021)). Upon inspection, we found that *upd2* was maximally transcribed ($\hat{x}$) in 4-6 hour old *D. melanogaster* embryos, which is also the same stage at which *hpo*, a known effector

of ovariole numbers in larval ovaries (Sarikaya and Extavour 2015) is reported as maximally transcribed (Li, et al. 2014). The 4-6 hour old embryos comprise an embryonic stage during which the somatic gonad precursors (SGPs) are being established (Richardson and Lehmann 2010), and thus speculatively these two genes (*upd2* and *hpo*) could potentially influence ovariole numbers by affecting SGP number or behaviour (King, et al. 1968; Sarikaya, et al. 2012; Sarikaya and Extavour 2015). In sum, *upd2* protein sequence changes may contribute to the divergence in ovariole numbers in the *melanogaster* subgroup, particularly the marked decline in the *D. sechellia* branch.

<u>*Zyxin (Zyx)*</u>

For the SIGNALC ovariole-related gene *Zyx*, which modulates activity of the Hippo pathway (table S3; Rauskolb et al. 2011; Gaspar et al. 2014; Ma et al. 2016), each of the five species of the *melanogaster* subgroup had a gene-wide dN/dS value (ranging from 0.267 up to >1, table 1) that was more than threefold higher than the genome-wide median per respective species branch (the genome-wide median dN/dS values for each of the five branches are shown in fig. S1). The patterns suggest potential functional divergence of *Zyx* throughout the clade. *Zyx* exhibited gene-wide dN/dS>1 in *D. sechellia*, suggesting a history of positive selection in that lineage, that co-occurred with its marked decline in ovariole numbers (fig. 2), suggesting the potential for a causative relationship. Upon close examination, we found that *Zyx* in the *D. sechellia* branch was a case when dS approached 0 (the other species branches had dS between 0.006 to 0.010), however the dN value was 0.004 (above the cut-off of 0.001), which was double the dN for *D. simulans* and *D. melanogaster* (even though dS was higher for the latter two species, their dN was lower), together indicating that the elevated dN/dS in *D. sechellia* was due to elevated dN (not low dS, and is conservatively denoted as dN/dS>1). Overall, the patterns suggest positive selection of *Zyx* in *D. sechellia*.

There are plausible cell biological mechanisms whereby adaptive changes in the *Zyx* protein may potentially influence ovariole number divergence. The gene *Zyx* encodes an actin cytoskeleton regulator, with roles in Hippo pathway regulation (table S3, (Rauskolb, et al. 2011)), and evidence suggests it can regulate cell behaviors and movements during development (Amsellem, et al. 2005; Matsui and Lai 2013). Given that actin cytoskeleton activity is a prime

candidate for coordinating the cell movements required for the formation of the TFs (Chen, et al. 2001), it may be speculated that Zyx may contribute to this process, and thereby changes in its amino acid sequence may affect TF formation (table 1), and in turn, alter the ovariole numbers (King, et al. 1968; Sarikaya, et al. 2012; Sarikaya and Extavour 2015). This provides a possible mechanism whereby functional changes in the Zyx protein may affect interspecies transitions in ovariole numbers. Further, *Zyx* positively regulates Yki (Rauskolb, et al. 2011), a transcriptional coactivator involved in many processes including cell proliferation (Huang, et al. 2005), and that accumulates in the nucleus in *hpo* RNAi larval ovary somatic cells (Sarikaya and Extavour 2015). We previously showed that elevating Yki levels in the somatic ovary results in an increased number of TFs and ovarioles (Sarikaya and Extavour 2015). Moreover, loss of *Zyx* reduces Yki activity (Rauskolb, et al. 2011). Thus, taken together, it may be hypothesized that the low ovariole numbers in *D. sechellia* as compared to all other *melanogaster* subgroup species (fig. 2) may have arisen from a reduction of Yki activity, that was caused by lowered function/activity of Zyx protein due to its amino acid sequence changes (table 1). Given that Z*yx* exhibited signs of positive selection in the *D. sechellia* branch (gene-wide dN/dS, table 1), this would in theory imply that the reduction in ovariole numbers (rather than an increase) in *D. sechellia* may comprise an adaptive reproductive change.

Phosphatase and tensin homolog (Pten)

The SIGNALC ovariole-related gene *Pten* (Kumar, et al. 2020) is a regulator of the mTOR and insulin signalling pathways (table S3). *Pten* exhibited an exceptionally high dN/dS value (0.594) in the *D. erecta* branch, more than five-fold higher than the *D. erecta* genome-wide median (0.104, fig. S1) and had branch-site positive selection (in *D. erecta*, $P<0.05$). This gene also exhibited a markedly elevated dN/dS value in the *D. melanogaster* branch (0.3122, table 1) and showed positive selection using the McDonald and Kreitman (1991) tests that were based on inter- versus intraspecies divergence solely for *D. melanogaster*-*D. simulans* ($P<0.012$, see Methods, and table 1 Notes (Murga-Moreno, et al. 2019)). *D. erecta* has an intermediate number of ovarioles per female (value of 27, fig. 2) among the five-species of the *melanogaster* subgroup (fig. 2) and forms an outgroup clade with *D. yakuba* (ovariole number 25.8). Similar to numerous other ovariole-related SIGNALC genes in table 1, *Pten* has core functions in regulating the actin

cytoskeleton (von Stein, et al. 2005) (table S3), and is involved in cell size, cell growth and tumor suppression (Goberdhan, et al. 1999). Mutations in *Pten* impair cytoskeletal activity in *Drosophila* ovaries, and induce various female reproductive phenotypes including impaired localization of the germ plasm components *oskar* mRNA and Vasa protein in laid eggs and an absence of pole cells (von Stein, et al. 2005). Further, *Pten* RNAi in the *D. melanogaster* larval ovaries increases ovariole number (the *hpo[RNAi]* Ovariole Number phenotype (Kumar, et al. 2020), see table 1). Among all of the 59 tissues/stages used for *tau* analyses (which was a relatively low value of 0.663 in *D melanogaster* for this gene), *Pten* had maximal expression, $\hat{x}$=1, for the adult virgin ovaries (table S3), which is also consistent with an essential female ovarian role. In sum, given its functions in female reproductive system, and the rapid evolution of this signalling gene in four of five of the *Drosophila* lineages (0.128-0.594), and explicit evidence of positive selection in *D. erecta* (table 1), we hypothesize that *Pten* is a putative factor involved in ovariole number transitions in the *melanogaster* subgroup, and that may shape ovariole numbers by adaptive evolution, particularly in the *D. erecta* branch (table 1).

vein (vn)

The ovariole-related EGF pathway ligand gene *vn* (table S3) exhibited a dN/dS value that was between 1.6 to 7.2 fold higher than the genome-wide median per respective species branch in three of the *Drosophila* species branches studied (*D. simulans*, *D. sechellia* and *D. yakuba* had values of 0.4712, 0.2069, and 0.2802 respectively; table 1; genome-wide medians in fig. S1), and presented signals of branch-site positive selection in the *D. yakuba* branch ($P<0.05$, table 1). *vn* is involved in growth and tissue patterning, including roles in the *D. melanogaster* adult ovary in somatic escort cells and in the differentiation of female germ stem cells (Slaidina, et al. 2021; Tu, et al. 2021). Further, this gene is expressed in the *D. melanogaster* TFs and SH cells (Slaidina, et al. 2020), which regulate ovariole formation and number (King, et al. 1968; Sarikaya, et al. 2012; Sarikaya and Extavour 2015; Slaidina, et al. 2020), consistent with the finding that *vn* RNAi reduces ovariole number in *D. melanogaster* (the *hpo[RNAi]* Ovariole Number phenotype, table 1 (Kumar, et al. 2020)). The highly elevated dN/dS value for *vn* in both *D. simulans* and *D. sechellia* terminal branches in particular suggest that *vn* protein sequence evolution may contribute to the rapid and two-fold level change in ovariole numbers between these closely related species (fig. 2).

In sum, *vn* has signs of evolvability in its protein sequence, with frequent protein sequence changes and adaptive evolution (in *D. yakuba*) and yet also exhibited relatively slow evolution in the *D. melanogaster* branch (dN/dS=0.0841), perhaps due to high pleiotropy (*tau*=0.741, table S3; note *tau* was measured in *D. melanogaster* as the reference), suggesting the gene has been subjected to strong purifying selection in that lineage. Thus, *vn* appears to have a dynamic evolutionary pattern, suggestive of potential roles in ovariole number divergence in the *D. simulans* and *D. sechellia* branches, and in *D. yakuba*.

BULKSG genes (table 2)

<u>*Insulin-like peptide 5 (Ipl5)*</u>

The ovariole-related gene *Ilp5* in table 2 was particularly notable in that it exhibited strong purifying selection in *D. melanogaster* (dN/dS=0.001) and had high dN/dS values in all other branches (values of 0.2932-0.5843 in *D. simulans*, *D. sechellia*, *D. yakuba*, *D. erecta*, each markedly higher than the genome-wide branch median values, which ranged from 0.066 to 0.125, fig. S1). It may be the case that gene redundancy has contributed to the divergence patterns for this gene. The *D. melanogaster Ilp* family has eight genes that have evolved a range of functions and tissue type specializations, including ovarian functions for *Ilp5* (Gronke, et al. 2010). In gene families, one gene member (or another) may pass through various stages of partial redundancy of function while still retaining the original gene function (Ohno 1970; Kirschner and Gerhart 1998), creating a window for relaxed constraint, and evolvability, of protein-coding regions and potentially giving rise to adaptive new functions or subfunctions, including gene-dosage functions (Kirschner and Gerhart 1998; Presgraves 2005; Conant and Wolfe 2008; Kuzmin, et al. 2022). Otherwise, without a function, the genes may accumulate mutations to form a pseudogene or become lost from the genome (Ohno 1970; Presgraves 2005; Conant and Wolfe 2008; Kuzmin, et al. 2022). Given the gene *Ilp5* was retained in the genomes of all five species here (table 2), it may be theorized that its rapid sequence divergence in four of five terminal branches may be associated with the evolution of altered functions that may affect ovariole formation. In fact, *llp5* has been linked to female re-mating frequency in *D. sechellia* (Wigby, et al. 2011), and thus sexual selection pressures could potentially contribute to its very high dN/dS in that branch (0.5843, table 2), and to the evolved decrease in ovariole numbers in this lineage (fig. 2). With respect to the

comparatively slow evolution of this gene in *D. melanogaster*, a study of *Ilp5* knockouts found evidence for compensatory expression of *Ilp3* (Gronke et al. 2010). *Ilp5* loss of function conditions cause a decrease in egg laying but do not completely eliminate it (Kumar, et al. 2020), suggesting its redundancy with other *Ilp* family members (and gene-dose compensation) may limit the extent of the negative effects on egg production. Nonetheless, the overall effect on egg yield and thus likely on fitness (Gronke, et al. 2010) may explain the strong purifying selection and low dN/dS value that we observed for the *D. melanogaster* branch, particularly if a weaker effect of *Ilp5* mutations occurred in the other species. In sum, *Ilp5* presents a dynamic history in the *melanogaster* subgroup and may substantively contribute to ovariole number variation in this taxon.

aquarius (*aqrs*)

The BULKSG ovariole-related gene *aquarius* (*aqrs*) (table 2) had the highest gene-wide dN/dS in the *D. yakuba* branch (0.3029), followed by *D. erecta* (0.1972), suggesting particularly rapid changes in the outgroup species (fig. 2). Aqrs protein has been associated with the functionality of the male seminal fluid proteins (SFPs) and the sex peptide (SP) in *D. melanogaster* (Findlay, et al. 2014), products that are transferred to the female reproductive tract during copulation and act to increase egg production and decrease female receptivity to courtship (Findlay, et al. 2014). The observed high levels of *aqrs* transcripts in larval somatic ovary cells (table 2) suggests that the gene also plays some role in these female reproductive cells. In this regard, *aqrs* may be a strong target for male-female sexual conflict, which is a factor known to cause rapid evolution of female (and male) proteins and of reproductive traits such as egg production, and may ultimately promote speciation (Rice 1996; Arnqvist, et al. 2000; Swanson and Vacquier 2002; Clark, et al. 2009). Thus, the rapid sequence divergence observed here for *aqrs* may putatively contribute to interspecies ovariole number variation.

SINGLEC Genes (table S7; fig. 4)

Ecdysone-inducible gene E1 (*ImpE1*), *sm*, and *CLIP-190*

In terms of the subset of rapidly evolving genes that we identified that were exclusively upregulated in TFs (TFa and/or TFp, and no other cell types, table S7 Notes), examples included *Ecdysone-inducible gene E1* (*ImpE1*), *smooth (sm*), and *Cytoplasmic linker protein 190* (*CLIP-190*), each of which exhibited positive selection in the *D. sechellia* branch (table S7). *ImpE1* has been linked to cell rearrangements during morphogenesis (Natzle, et al. 1988), *sm* is a nuclear ribonucleoprotein involved in diverse roles including muscle functions (Draper, et al. 2009), neuronal and chemosensory processes (Layalle, et al. 2005), and *CLIP-190* proteins coordinate binding between actins and microtubules (Lantz and Miller 1998), and may affect intracellular transport (Sanghavi, et al. 2012). Taken together, the rapidly evolving genes upregulated in TF cell types are involved in multiple cellular processes likely required for TF morphogenesis, and thus adaptive changes observed in these genes (fig. 4) may contribute to the proximate mechanisms underlying the rapid evolutionary divergence in ovariole numbers in *Drosophila*.

Genes downregulated in the BULKSG soma cells versus germ cells (table S7)

In terms of genes upregulated in the germ cells (and thus downregulated in the soma), the top ten genes with the highest degree of upregulation are shown in table S5. The genes exhibited elevated *tau* values ranging from 0.880 to 0.975, indicating a tendency of narrow transcription breadth (and several very narrow, with *tau* values above 0.950) for these genes, potentially reflecting high specialization to germ cell functions. Further, 8 of the 10 genes were largely uncharacterized genes (annotations as "CG number" only in table S5) based on available annotation from DAVID (Huang da, et al. 2009) and FlyBase (Gramates, et al. 2022). The two relatively well characterized genes *no long nerve cord* (*nolo*) and *orientation disruptor* (*ord*) play roles in the nerve cord, extracellular matrix, and meiosis (Huang da, et al. 2009). As the evidence to date suggests that somatic cells, rather than germ cells, are the principal regulators of ovariole number, we did not focus further on this particular genes of very highly and positively upregulated germ cell genes, and instead focused in the main text on the genes upregulated in somatic cells and with variable expression across larval ovary stages in tables 3 and 4.

BULKSG for three stages of larval ovary development

We also identified genes from the BULKSG dataset with respect to the data for three stages of larval ovary soma development (early, mid or late). The 30 genes with the highest degree of upregulation (log_2 change) in one stage (versus the other two) and that also exhibited rapid evolution (M0 dN /dS>0.20) are provided in table S6 (given the three types of comparisons, we included the top 30 genes, rather than ten as in tables 2, table S5). These genes were utilized in conjunction with sc-RNA (SINGLEC) in table 3.

Supplementary Results For The Three-Species Hawaiian *Drosophila* Clade

Table S10 contains the dN/dS results for the Hawaiian *Drosophil*a orthologs of the 27 SIGNALC genes identified in table 1 from the *melanogaster* subgroup (Kumar, et al. 2020), and are described in the main text. Here, we note that six of the 27 studied genes of interest from the *melanogaster* subgroup (from table 1) had high confidence orthologs (reciprocal BLASTX) identified between *D. melanogaster-D. grimshawi* but not among all three species of *D. murphyi-D. sproati-D. grimshawi*. This may reflect rapid evolution of these genes in Hawaiian *Drosophila*, gene loss from the genome, and/or genes missing from the annotation (or lacking a complete CDS that were removed), although the latter has been largely mitigated given the high BUSCO scores from each genome (see the Supplementary Methods section above). Nonetheless, the data in table S10 infers rapid evolution of many of the SIGNALC genes (table S10), including positive selection, in the Hawaiian taxa (for those that had high confidence orthologs).

The ovariole related genes identified from the SINGLEC dataset in fig. 4 (and fig. S4, table S7), are less apt to share functions in Hawaiian *Drosophila* (than SIGNALC) given the fast evolution of gene expression of reproductive genes, including in *Drosophila* (Ranz, et al. 2003; Whittle and Extavour 2019). Thus, we excluded analyses of dN/dS and branch-site positive selection for this group pending future data on sc-RNA sequence or bulk-RNA seq of those cell types in *D. grimshawi* or other closely related Hawaiian taxa. Nonetheless, as cited in the main text, the genes that were highly upregulated in the *D. melanogaster* TF and SH cells (fig. 4, fig. S4, N values therein), were found to have the lowest frequency of high confidence ortholog gene sets identified in Hawaiian *Drosophila* among the nine cell types. The genes without orthologs included those from the *D. grimshawi-D. melanogaster* genome contrast (44.6% of all studied

genes in fig. 4without Hawaiian orthologs were from this class) and from the genome contrasts among the three Hawaiian species (55.4% of all studied genes without Hawaiian orthologs were from this class, fig. S6). This pattern suggests that the genes upregulated in the TF and SH cells (in *D. melanogaster*) as a group have evolved rapidly across the studied *Drosophila* species making orthologs unrecognizable, and/or have more commonly exhibited gene gains and losses, than genes from other cell types (Tautz and Domazet-Loso 2011; Tautz, et al. 2013), as described in the main text.

Supplementary References

Altschul SF, Madden TL, Schaffer AA, Zhang J, Zhang Z, Miller W, Lipman DJ. 1997. Gapped BLAST and PSI-BLAST: a new generation of protein database search programs. Nucleic Acids Research 25:3389-3402.

Amsellem V, Kryszke MH, Hervy M, Subra F, Athman R, Leh H, Brachet-Ducos C, Auclair C. 2005. The actin cytoskeleton-associated protein zyxin acts as a tumor suppressor in Ewing tumor cells. Experimental Cell Research 304:443-456.

Arnqvist G, Edvardsson M, Friberg U, Nilsson T. 2000. Sexual conflict promotes speciation in insects. Proceedings of the National Academy of Sciences of the United States of America 97:10460-10464.

Assis R, Zhou Q, Bachtrog D. 2012. Sex-biased transcriptome evolution in Drosophila. Genome Biology and Evolution 4:1189-1200.

Castresana J. 2000. Selection of conserved blocks from multiple alignments for their use in phylogenetic analysis. Mol Biol Evol 17:540-552.

Chen J, Godt D, Gunsalus K, Kiss I, Goldberg M, Laski FA. 2001. Cofilin/ADF is required for cell motility during *Drosophila* ovary development and oogenesis. Nature Cell Biology 3:204-209.

Clark NL, Gasper J, Sekino M, Springer SA, Aquadro CF, Swanson WJ. 2009. Coevolution of interacting fertilization proteins. PLoS Genetics 5:e1000570.

Conant GC, Wolfe KH. 2008. Turning a hobby into a job: how duplicated genes find new functions. Nature Reviews: Genetics 9:938-950.

Cui Q, Purisima EO, Wang E. 2009. Protein evolution on a human signaling network. BMC Systems Biology 3:21.

Darfour-Oduro KA, Megens HJ, Roca AL, Groenen MA, Schook LB. 2016. Evolutionary patterns of Toll-like receptor signaling pathway genes in the Suidae. BMC Evolutionary Biology 16:33.

Draper I, Tabaka ME, Jackson FR, Salomon RN, Kopin AS. 2009. The evolutionarily conserved RNA binding protein SMOOTH is essential for maintaining normal muscle function. Fly (Austin) 3:235-246.

Edgar RC. (r20416 co-authors). 2004. MUSCLE: a multiple sequence alignment method with reduced time and space complexity. BMC Bioinformatics 5:113.

Findlay GD, Sitnik JL, Wang W, Aquadro CF, Clark NL, Wolfner MF. 2014. Evolutionary rate covariation identifies new members of a protein network required for *Drosophila* melanogaster female post-mating responses. PLoS Genetics 10:e1004108.

Goberdhan DC, Paricio N, Goodman EC, Mlodzik M, Wilson C. 1999. *Drosophila* tumor suppressor PTEN controls cell size and number by antagonizing the Chico/PI3-kinase signaling pathway. Genes Dev 13:3244-3258.

Gramates LS, Agapite J, Attrill H, Calvi BR, Crosby MA, Dos Santos G, Goodman JL, Goutte-Gattat D, Jenkins VK, Kaufman T, et al. 2022. FlyBase: a guided tour of highlighted features. Genetics 220.

Gramates LS, Marygold SJ, Santos GD, Urbano JM, Antonazzo G, Matthews BB, Rey AJ, Tabone CJ, Crosby MA, Emmert DB, et al. 2017. FlyBase at 25: looking to the future. Nucleic Acids Research 45:D663-D671.

Gronke S, Clarke DF, Broughton S, Andrews TD, Partridge L. 2010. Molecular evolution and functional characterization of *Drosophila* insulin-like peptides. PLoS Genetics 6:e1000857.

Hoff KJ, Stanke M. 2013. WebAUGUSTUS--a web service for training AUGUSTUS and predicting genes in eukaryotes. Nucleic Acids Research 41:W123-128.

Holt C, Yandell M. 2011. MAKER2: an annotation pipeline and genome-database management tool for second-generation genome projects. BMC Bioinformatics 12:491.

Huang da W, Sherman BT, Lempicki RA. 2009. Systematic and integrative analysis of large gene lists using DAVID bioinformatics resources. Nature protocols 4:44-57.

Huang J, Wu S, Barrera J, Matthews K, Pan D. (p07161 co-authors). 2005. The Hippo signaling pathway coordinately regulates cell proliferation and apoptosis by inactivating Yorkie, the *Drosophila* Homolog of YAP. Cell 122:421-434.

Kim BY, Wang JR, Miller DE, Barmina O, Delaney E, Thompson A, Comeault AA, Peede D, D'Agostino ER, Pelaez J, et al. 2021. Highly contiguous assemblies of 101 drosophilid genomes. Elife 10.

King RC, Aggarwal SK, Aggarwal U. 1968. The development of the female *Drosophila* reproductive system. Journal of Morphology 124:143-166.

Kirschner M, Gerhart J. 1998. Evolvability. Proceedings of the National Academy of Sciences of the United States of America 95:8420-8427.

Kumar S, Stecher G, Tamura K. 2016. MEGA7: Molecular Evolutionary Genetics Analysis Version 7.0 for Bigger Datasets. Molecular Biology and Evolution 33:1870-1874.

Kumar T, Blondel L, Extavour CG. 2020. Topology-driven protein-protein interaction network analysis detects genetic sub-networks regulating reproductive capacity. Elife 9.

Kuzmin E, Taylor JS, Boone C. 2022. Retention of duplicated genes in evolution. Trends in Genetics 38:59-72.

Lantz VA, Miller KG. 1998. A class VI unconventional myosin is associated with a homologue of a microtubule-binding protein, cytoplasmic linker protein-170, in neurons and at the posterior pole of *Drosophila* embryos. Journal of Cell Biology 140:897-910.

Larracuente AM, Sackton TB, Greenberg AJ, Wong A, Singh ND, Sturgill D, Zhang Y, Oliver B, Clark AG. 2008. Evolution of protein-coding genes in *Drosophila*. Trends in Genetics 24:114-123.

Layalle S, Coessens E, Ghysen A, Dambly-Chaudiere C. 2005. Smooth, a hnRNP encoding gene, controls axonal navigation in *Drosophila*. Genes to Cells 10:119-125.

Li JJ, Huang H, Bickel PJ, Brenner SE. 2014. Comparison of *D. melanogaster* and *C. elegans* developmental stages, tissues, and cells by modENCODE RNA-seq data. Genome Research 24:1086-1101.

Lobell AS, Kaspari RR, Serrano Negron YL, Harbison ST. 2017. The Genetic Architecture of Ovariole Number in *Drosophila melanogaster*: Genes with Major, Quantitative, and Pleiotropic Effects. G3 (Bethesda) 7:2391-2403.

Love MI, Huber W, Anders S. 2014. Moderated estimation of fold change and dispersion for RNA-seq data with DESeq2. Genome Biology 15:550.

Mank JE, Ellegren H. 2009. Are sex-biased genes more dispensable? Biology letters 5:409-412.

Manni M, Berkeley MR, Seppey M, Simao FA, Zdobnov EM. 2021. BUSCO Update: Novel and Streamlined Workflows along with Broader and Deeper Phylogenetic Coverage for Scoring of Eukaryotic, Prokaryotic, and Viral Genomes. Mol Biol Evol 38:4647-4654.

Masalia RR, Bewick AJ, Burke JM. 2017. Connectivity in gene coexpression networks negatively correlates with rates of molecular evolution in flowering plants. PloS One 12:e0182289.

Matsui Y, Lai ZC. 2013. Mutual regulation between Hippo signaling and actin cytoskeleton. Protein Cell 4:904-910.

McDonald JH, Kreitman M. 1991. Adaptive protein evolution at the Adh locus in Drosophila. Nature 351:652-654.

Meisel RP. 2011. Towards a more nuanced understanding of the relationship between sex-biased gene expression and rates of protein-coding sequence evolution. Molecular Biology and Evolution 28:1893-1900.

Murga-Moreno J, Coronado-Zamora M, Hervas S, Casillas S, Barbadilla A. 2019. iMKT: the integrative McDonald and Kreitman test. Nucleic Acids Research 47:W283-W288.

Natzle JE, Fristrom DK, Fristrom JW. 1988. Genes expressed during imaginal disc morphogenesis: IMP-E1, a gene associated with epithelial cell rearrangement. Developmental Biology 129:428-438.

Ohno S. 1970. Evolution by gene duplication. New York: Springer Verlag.

Otto SP. 2004. Two steps forward, one step back: the pleiotropic effects of favoured alleles. Proceedings: Biological Sciences 271:705-714.

Presgraves DC. 2005. Evolutionary genomics: new genes for new jobs. Current Biology 15:R52-53.

Ranz JM, Castillo-Davis CI, Meiklejohn CD, Hartl DL. 2003. Sex-dependent gene expression and evolution of the *Drosophila* transcriptome. Science 300:1742-1745.

Rauskolb C, Pan G, Reddy BV, Oh H, Irvine KD. 2011. Zyxin links fat signaling to the hippo pathway. PLoS Biology 9:e1000624.

Rice WR. 1996. Sexually antagonistic male adaptation triggered by experimental arrest of female evolution. Nature 381:232-234.

Richardson BE, Lehmann R. 2010. Mechanisms guiding primordial germ cell migration: strategies from different organisms. Nature Reviews: Molecular Cell Biology 11:37-49.

Sanghavi P, Lu S, Gonsalvez GB. 2012. A functional link between localized Oskar, dynamic microtubules, and endocytosis. Developmental Biology 367:66-77.

Sarikaya DP, Belay AA, Ahuja A, Dorta A, Green DA, 2nd, Extavour CG. 2012. The roles of cell size and cell number in determining ovariole number in *Drosophila*. Developmental Biology 363:279-289.

Sarikaya DP, Church SH, Lagomarsino LP, Magnacca KN, Montgomery SL, Price DK, Kaneshiro KY, Extavour CG. 2019. Reproductive Capacity Evolves in Response to Ecology through Common Changes in Cell Number in Hawaiian Drosophila. Current Biology 29:1877-1884.e1876.

Sarikaya DP, Extavour CG. 2015. The Hippo pathway regulates homeostatic growth of stem cell niche precursors in the *Drosophila* ovary. PLoS Genetics 11:e1004962.

Satija R, Farrell JA, Gennert D, Schier AF, Regev A. 2015. Spatial reconstruction of single-cell gene expression data. Nature Biotechnology 33:495-502.

Seppey M, Manni M, Zdobnov EM. 2019. BUSCO: Assessing Genome Assembly and Annotation Completeness. Methods in Molecular Biology 1962:227-245.

Slaidina M, Banisch TU, Gupta S, Lehmann R. 2020. A single-cell atlas of the developing Drosophila ovary identifies follicle stem cell progenitors. Genes Dev 34:239-249.

Slaidina M, Gupta S, Banisch TU, Lehmann R. 2021. A single-cell atlas reveals unanticipated cell type complexity in *Drosophila* ovaries. Genome Research 31:1938-1951.

Stanke M, Waack S. 2003. Gene prediction with a hidden Markov model and a new intron submodel. Bioinformatics 19 Suppl 2:ii215-225.

Stanley CE, Jr., Kulathinal RJ. 2016. flyDIVaS: A Comparative Genomics Resource for Drosophila Divergence and Selection. G3 (Bethesda) 6:2355-2363.

Starmer W, Polak M, Pitnick S, McEvey S, Barke rJ, Wolf L. 2003. Phylogenetic, Geographical, and Temporal Analysis of Female Reproductive Trade-Offs in Drosophilidae. Evolutionary Biology 33:139-171.

Suvorov A, Kim BY, Wang J, Armstrong EE, Peede D, D'Agostino ERR, Price DK, Waddell P, Lang M, Courtier-Orgogozo V, et al. 2022. Widespread introgression across a phylogeny of 155 *Drosophila* genomes. Current Biology 32:111-123 e115.

Swanson WJ, Vacquier VD. 2002. The rapid evolution of reproductive proteins. Nature Reviews: Genetics 3:137-144.

Talavera G, Castresana J. (r20429 co-authors). 2007. Improvement of phylogenies after removing divergent and ambiguously aligned blocks from protein sequence alignments. Systematic Biology 56:564-577.

Tarikere S, Ylla G, Extavour CG. 2022. Distinct gene expression dynamics in germ line and somatic tissue during ovariole morphogenesis in *Drosophila melanogaster*. G3 (Bethesda) 12: DOI: 10.1093/g1093journal/jkab1305

Tautz D, Domazet-Loso T. 2011. The evolutionary origin of orphan genes. Nature Reviews Genetics 12:692-702.

Tautz D, Neme R, Domazet-Loso T. 2013. Evolutionary Origin of Orphan Genes. eLS.

Tu R, Duan B, Song X, Chen S, Scott A, Hall K, Blanck J, DeGraffenreid D, Li H, Perera A, et al. 2021. Multiple Niche Compartments Orchestrate Stepwise Germline Stem Cell Progeny Differentiation. Current Biology 31:827-839 e823.

von Stein W, Ramrath A, Grimm A, Muller-Borg M, Wodarz A. 2005. Direct association of Bazooka/PAR-3 with the lipid phosphatase PTEN reveals a link between the PAR/aPKC complex and phosphoinositide signaling. Development 132:1675-1686.

Whittle CA, Extavour CG. 2019. Selection shapes turnover and magnitude of sex-biased expression in *Drosophila* gonads. BMC Evolutionary Biology 19:60 (doi: 10.1186/s12862-12019-11377-12864).

Whittle CA, Kulkarni A, Extavour CG. 2021. Evolutionary dynamics of sex-biased genes expressed in cricket brains and gonads. Journal of Evolutionary Biology 34:1188-1211.

Wigby S, Slack C, Gronke S, Martinez P, Calboli FC, Chapman T, Partridge L. 2011. Insulin signalling regulates remating in female *Drosophila*. Proceedings: Biological Sciences 278:424-431.

Yang Z. 2007. PAML 4: phylogenetic analysis by maximum likelihood. Molecular Biology and Evolution 24:1586-1591.
